# Organic binary charge-transfer compounds of 2,2′ : 6′,2′′ : 6′′,6-trioxotriphenylamine and a pyrene-annulated azaacene as donors†

**DOI:** 10.1039/d2ra07322f

**Published:** 2023-01-30

**Authors:** Rajorshi Das, Michael Linseis, Stefan M. Schupp, Franciska S. Gogesch, Lukas Schmidt-Mende, Rainer F. Winter

**Affiliations:** a Fachbereich Chemie, Universität Konstanz Universitätsstrasse 10, 78457 Konstanz Germany rainer.winter@uni-konstanz.de; b Fachbereich Physik, Universität Konstanz Universitätsstrasse 10, 78457 Konstanz Germany

## Abstract

Three binary charge-transfer (CT) compounds resulting from the donor 2,2′ : 6′,2′′ : 6′′,6-trioxotriphenylamine (TOTA) and the acceptors F_4_TCNQ and F_4_BQ and of a pyrene-annulated azaacene (PAA) with the acceptor F_4_TCNQ are reported. The identity of these CT compounds are confirmed by single-crystal X-ray diffraction as well as by IR, UV-vis-NIR and EPR spectroscopy. X-ray diffraction analysis reveals a 1 : 1 stoichiometry for TOTA·F_4_TCNQ, a 2 : 1 donor : acceptor ratio in (TOTA)_2_·F_4_BQ, and a rare 4 : 1 stoichiometry in (PAA)_4_·F_4_TCNQ, respectively. Metrical parameters of the donor (D) and acceptor (A) constituents as well as IR spectra indicate full CT in TOTA·F_4_TCNQ, partial CT in (TOTA)_2_·F_4_BQ and only a very modest one in (PAA)_4_·F_4_TCNQ. Intricate packing motifs are present in the crystal lattice with encaged, π-stacked (F_4_TCNQ^−^)_2_ dimers in TOTA·F_4_TCNQ or mixed D/A stacks in the other two compounds. Their solid-state UV-vis-NIR spectra feature CT transitions. The CT compounds with F_4_TCNQ are electrical insulators, while (TOTA)_2_·F_4_BQ is weakly conducting.

## Abstract 2

Three binary charge-transfer (CT) compounds resulting from the donor 2,2′ : 6′,2′′ : 6′′,6-trioxotriphenylamine (TOTA) and the acceptors F_4_TCNQ and F_4_BQ and of a pyrene-annulated azaacene (PAA) with the acceptor F_4_TCNQ are reported.

## Graphical abstract

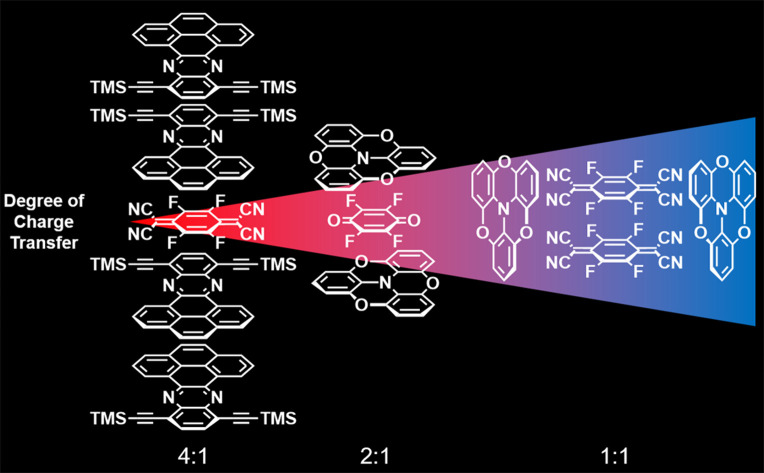

## Introduction

**Fig. 1:**
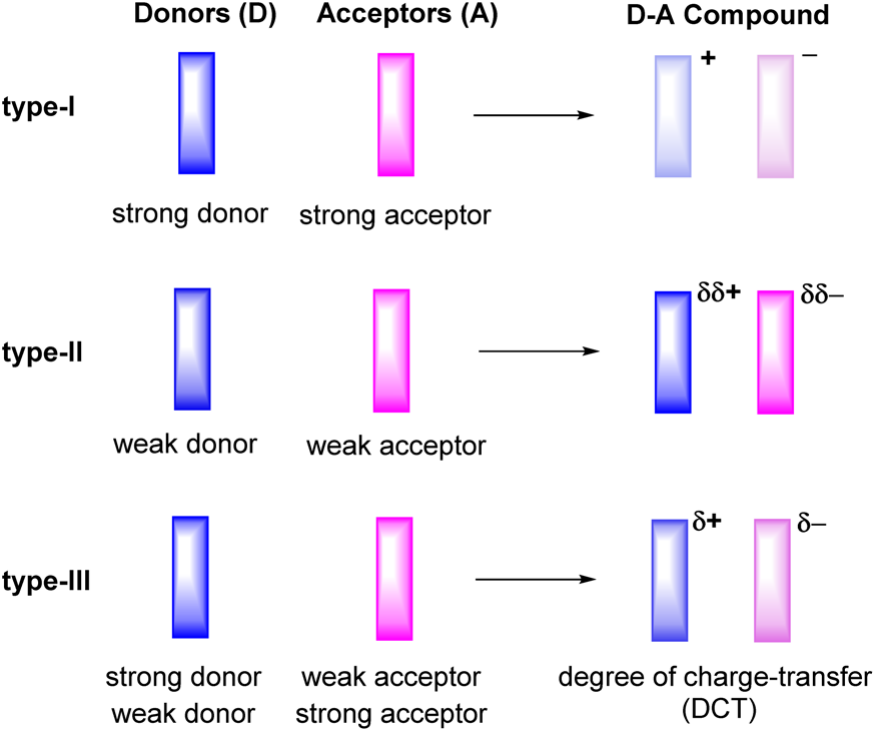
Degrees of charge-transfer for varieties of donor–acceptor (D–A) based organic CT compounds.

Ever since the first report on the charge-transfer compound TTF^+^·TCNQ^−^ (TTF = tetrathiafulvalene, TCNQ = tetracyanoquinodimethane) with metal-like conductivity,^1^ organic charge-transfer (CT) systems D^δ+^·A^δ−^, composed of a donor (D) and an acceptor (A), have attracted a great deal of attention.^2–6^ Compounds of this type can act as good electrical conductors or components of ambipolar semiconductors^3^ and may show other intriguing properties including strong electron–phonon coupling,^7,8^ photoelectricity,^9–13^ ferromagnetism,^14^ antiferromagnetism,^15^ or luminescence.^16–19^ The degree of charge-transfer between the D and A components tends to increase with a decreasing energy difference between the lowest unoccupied molecular orbital LUMO of the acceptor A and the highest occupied molecular orbital HOMO of the donor D. Such information can be retrieved by quantum chemical calculations^20^ or, on an experimental basis, from their electrochemical reduction or oxidation potentials, respectively.^3^ These data therefore provide useful guidelines to adjust the likely degree of CT and aid in the purposeful design of CT compounds. For example, the combination of a strong donor with a strong acceptor usually leads to the formation of a CT compound D^+^·A^−^ with a full transfer of charge (type-I, Fig. 1). This class of compounds may show strong ferromagnetism, but is often associated with only poor charge-transport capabilities.^15,21,22^

**Fig. 2:**
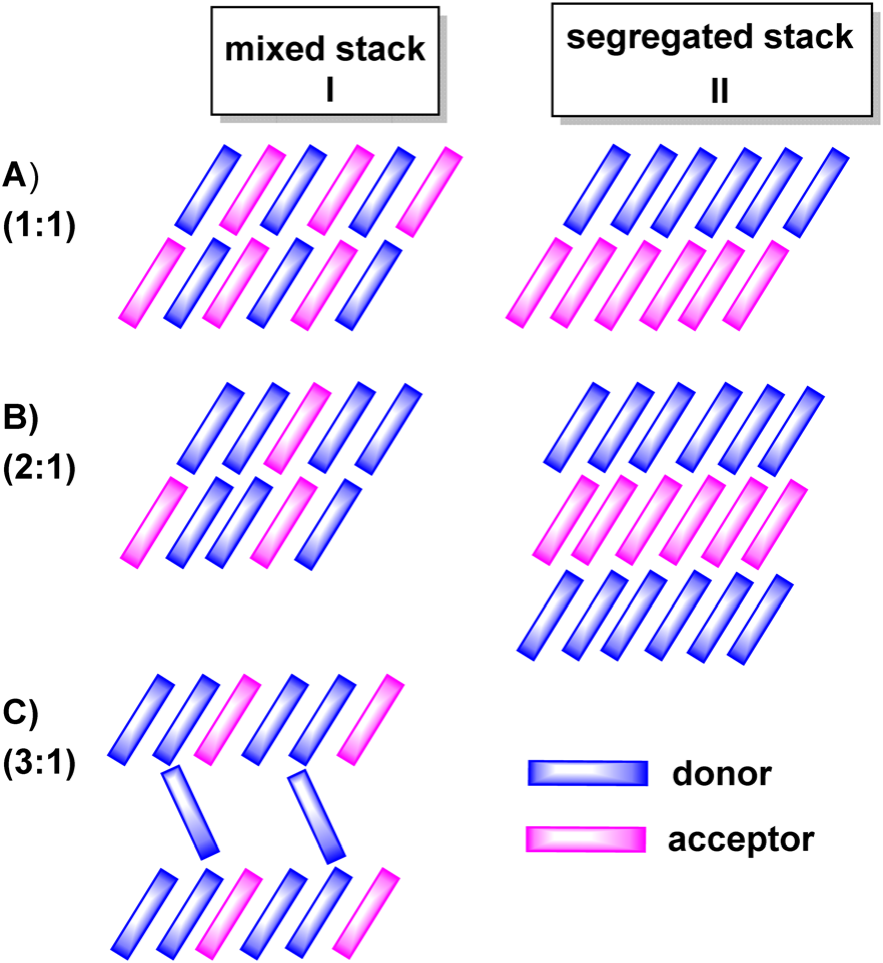
Different stoichiometric ratios (top to bottom) and stacking motifs (left to right) commonly observed in charge-transfer (CT) compounds. (A) 1 : 1, (B) 2 : 1 and (C) 3 : 1 donor/acceptor or acceptor/donor ratios. Column I displays structures with mixed stacks and column II structures with segregated stacks.

The properties of D–A-based CT compounds do, however, not only depend on the degree of charge-transfer, but also on the stoichiometric ratio D : A and on intermolecular D/D, A/A and D/A interactions in the crystal lattice. The various modes of π-stacking play a particularly important role in this respect.^3–5^ From the wealth of previous studies it has emerged that D–A based CT compounds where the donors and the acceptors form segregated stacks (motif II in Fig. 2) show often higher charge mobilities than CT compounds where the donors and the acceptors form alternating (1 : 1 ratio) or mixed stacks (*n* : 1 ratios) (motif I in Fig. 2). Illustrative examples are provided by various CT compounds assembled from TTF or its derivatives and TCNQ that crystallize in segregated D and A stacks and show conductivities of as high as 200 to 1000 S·cm^−1^.^3,21^ In contrast, CT compounds of tetramethoxyselenanthrenes and TCNQ form an alternately mixed-stacked structure [D⋯A]_∞_ of type A–I and exhibit poor conductivities of 4 × 10^−10^ S cm^−1^.^3,21^

**Fig. 3:**
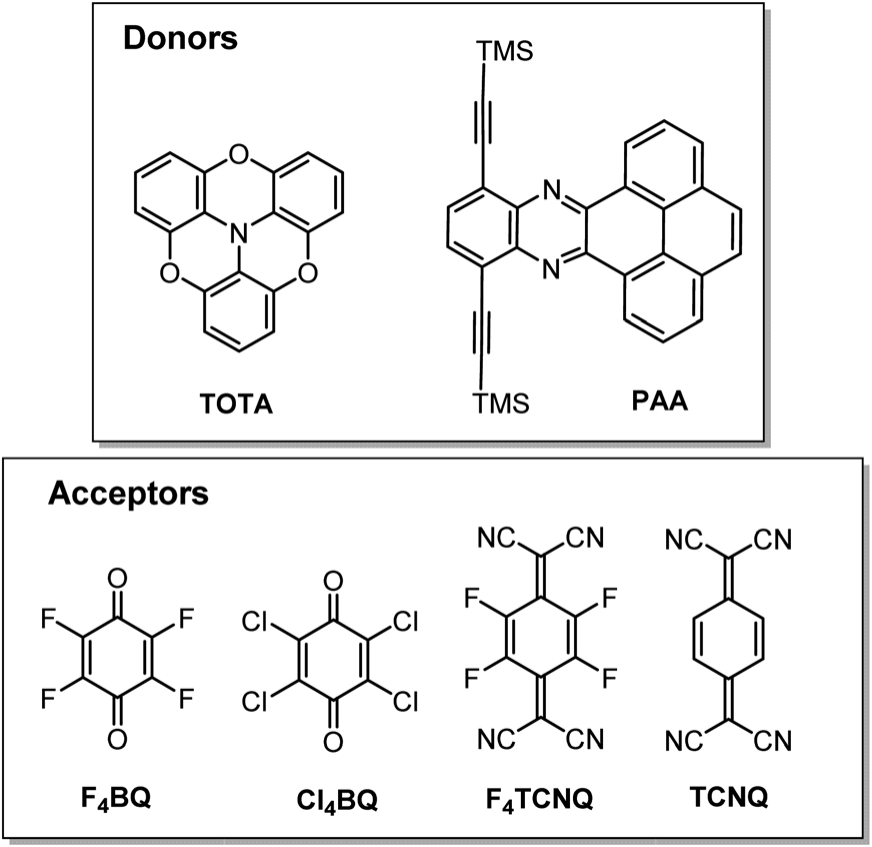
The donors and acceptors used in this study.

In this work, we have studied D–A compounds formed by combining the donors 2,2′ : 6′,2′′ : 6′′,6-trioxotriphenylamine (TOTA, Fig. 3) and a pyrene-annulated azaacene (PAA) with five different acceptors, namely the 2,3,5,6-tetrahalogeno-*p*-benzoquinones X_4_BQ (X = F, Cl, Br), 2,3,5,6-tetrafluoro-7,7,8,8-tetracyanoquinodimethane (F_4_TCNQ) and 7,7,8,8-tetracyanoquinodimethane (TCNQ). The donors constitute extended, planar π-systems and hence should be well-suited for π-stacking,^23–25^ but also allow for interdonor hydrogen bonding involving the O or N heteroatoms and for C≡N or C–F π-hole tetrel interactions.^26–30^ TOTA is a nonplanar, electron-rich molecule with a low half-wave potential *E*_1/2_^0/+^ of 110 mV (in CH_2_Cl_2_/NBu_4_PF_6_ against the ferrocene/ferrocenium standard couple FcH/FcH^+^ = 0 mV) for its first one-electron oxidation. In contrast, PAA shows its first oxidation potential at *E*_ox_^0/+^ = 1205 mV under the same experimental conditions and is hence a much weaker donor (Table 1, *vide infra*). The acceptors were chosen in order to cover a wider range of half-wave potentials for their first one-electron reduction, ranging from *E*_1/2_^0/−^ = 153 mV for F_4_TCNQ to −464 mV for Cl_4_BQ in the order F_4_TCNQ > TCNQ > F_4_BQ ≈ Cl_4_BQ ≈ Br_4_BQ (Table 1). We show that the degree of CT in the TOTA compounds varies considerably from the strong acceptor F_4_TCNQ to the weaker acceptor F_4_BQ as it is, *inter alia*, manifested by a structural change of the TOTA donor from bowl-shaped to planar with an increasing degree of CT. Almost no CT to F_4_TCNQ was observed for the CT compound with the weaker PAA donor.

**Table 1:** Cyclic voltammetry data of the donors and the acceptors of this study^a^

	*E*_1/2_^0/+^	*E*_1/2_^+/2+^	*E*_1/2_^0/−^	*E*_1/2_^−/2-^
TOTA	110	1250	—	—
PAA	1205^b^	—	−1590	—
F_4_TCNQ	—	—	153	−484
TCNQ	—	—	−270	−850
F_4_BQ	—	—	−448	−1336
Cl_4_BQ	—	—	−452	−1236
Br_4_BQ			−464	−1224

a All data in millivolts *versus* FcH/FcH^+^ in CH_2_Cl_2_/NBu_4_PF_6_ at r. t. and at *ν* = 100 mV s^−1^.

b Peak potential of the forward peak of a chemically irreversible anodic wave.

## Results and discussion

### Synthesis of the charge-transfer compounds

The D–A compounds formed on dissolving equimolar quantities of the respective pair of a donor and an acceptor in CH_2_Cl_2_ in a small vial. The vial was loosely capped by a screwcap and the solvent was allowed to slowly evaporate. After one or two weeks, dark purple crystals of a CT compound of TOTA and F_4_TCNQ, dark orange crystals of a TOTA/F_4_BQ and dark green crystals of a PAA/F_4_TCNQ D–A compound were obtained. Other combinations of one of these donors and another acceptor as well as using toluene as the solvent did not result in crystalline or phase-pure materials, although the formation of some pale-greenish crystals of a CT adduct of TOTA and TCNQ and of few yellow-green crystals from the PAA/TCNQ mixture were observed. These crystals did however not diffract well and could not be isolated in sufficient quantities and purity from the cocrystallized donor or acceptor to allow for their further investigation. Photographs of the three CT compounds discussed in this study and their precursors are provided in Fig. S1 in the ESI.†

As discussed in the introduction, the electron donating and accepting abilities of all donors and acceptors were investigated by cyclic voltammetry. Representative cyclic voltammograms are provided in Fig. S2 to S4 in the ESI†; relevant data are listed in Table 1.

### The TOTA-based CT compounds (TOTA)_2_·(F_4_TCNQ)_2_·CH_2_Cl_2_ and (TOTA)_2_·(F_4_BQ)

**Fig. 4:**
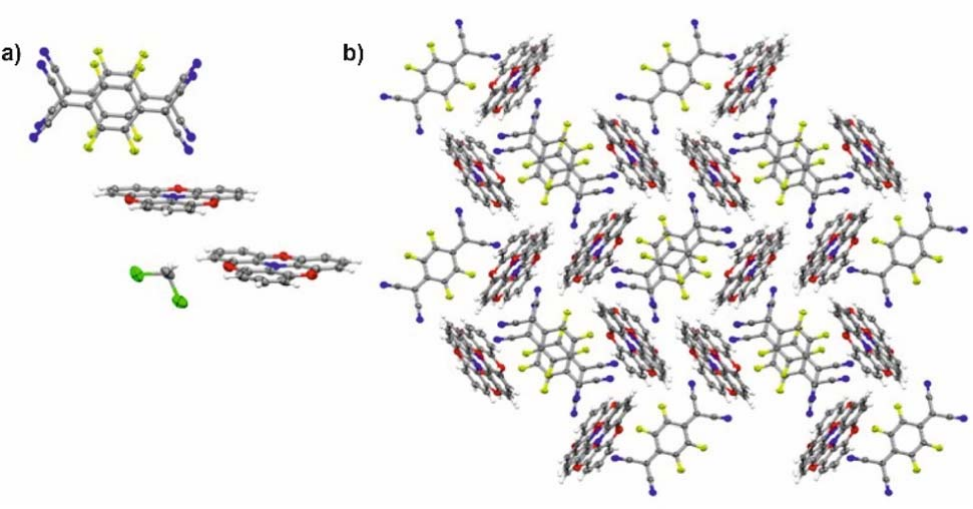
X-ray structure of (TOTA)_2_·(F_4_TCNQ)_2_·CH_2_Cl_2_. (a) Asymmetric unit showing the (F_4_TCNQ^−^)_2_ dimer and the two crystallographically different TOTA^+^ cations. (b) Packing motif of the donor and acceptor constituents in the crystal lattice; the CH_2_Cl_2_ solvent molecules are removed for clarity reasons. Colour codes: H, white; C, grey; N, blue; F, yellow-green; O, red; Cl, green.

2,2′ : 6′,2′′ : 6′′,6-Trioxotriphenylamine (TOTA) is slightly bowl-shaped and can be easily oxidized to a stable, planar radical cation.^31,32^ Its first oxidation potential *E*_1/2_^0/+^ of 110 mV against the ferrocene/ferrocenium standard (FcH/FcH^+^) in CH_2_Cl_2_/^*n*^Bu_4_PF_6_ (^*n*^Bu_4_PF_6_ = tetrabutylammonium hexafluorophosphate) is close to the reported value of 140 mV in DMF.^31^ Its reaction with equimolar quantities of the strong acceptor F_4_TCNQ accordingly yielded the 1 : 1 salt (TOTA)_2_·(F_4_TCNQ)_2_·CH_2_Cl_2_ with an essentially full transfer of charge between the donor and the acceptor (*vide infra*). The compound crystallized in the monoclinic space group *P*2_1_/*c* with two crystallographically distinct molecules of the donor and the acceptor each and one CH_2_Cl_2_ solvent molecule in the unit cell. The structure is shown in Fig. 4.

**Fig. 5:**
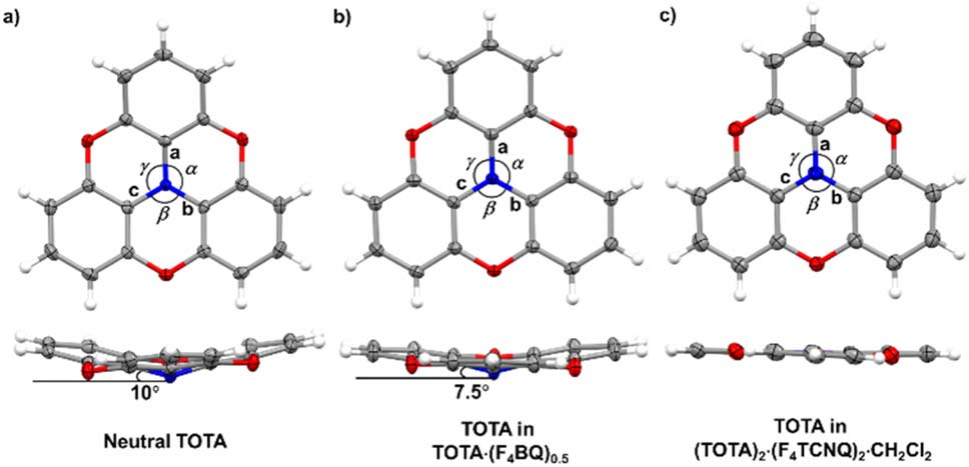
X-ray structures and metric parameters of (a) neutral TOTA,^31^ (b) the TOTA constituent in (TOTA)_2_·F_4_BQ and (c) the TOTA^+^ cation.

The bond parameters of F_4_TCNQ and, to a lesser extent, of TOTA are sensitive to their oxidation state. Neutral F_4_TCNQ has a quinoid structure with pronounced short-long-short bond length alternation (see Fig. 3 and Table 2). One-electron reduction increases the aromaticity of the central ring and renders the intracyclic CC bonds more similar while lengthening the exocyclic C=C bonds. This bond lengthening appears to constitute the most indicative structure change in tetracyanoquinodimethanes concomitant with reduction, which complies with the notion that the cyano groups are the primary electron acceptors.^46–48^ On the other hand, bond length changes on oxidation of TOTA are diluted over the entire polycyclic π-system so that the most indicative structural changes are the shortening of the N–C bonds and the flattening of the cone at the amine N atom from 10° to fully planar (see Fig. 5).

**Table 2:** Selected bond parameters of the CT compounds of this study and pertinent reference systems

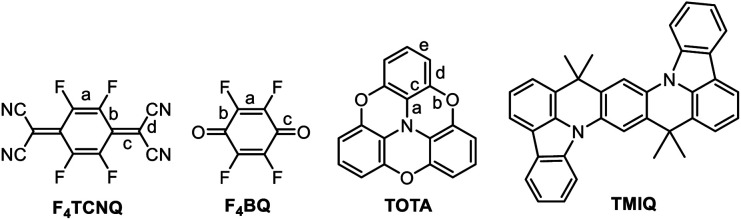						
	*a*	*b*	*c*	*d*	*e*	ref.
F_4_TCNQ^a^	1.337	1.439	1.372	1.437		33–36
F_4_TCNQ^−^a	1.358	1.417	1.418	1.430	1.385	37 and 38
TOTA	1.408	1.392	1.388	1.384	1.385	31
TOTA^+^^a^	1.376	1.375	1.394	1.378	1.378	31 and 32
F_4_BQ^a^	1.339	1.477	1.213	—	—	39–41
Cl_4_BQ	1.344	1.489	1.211	—	—	42
Cl_4_BQ^−^	1.360	1.448	1.248	—	—	43
TOTA·F_4_TCNQ^b^						This work
TOTA	1.376	1.375	1.397	1.397	1.389	
F_4_TCNQ	1.358	1.417	1.410	1.423	—	
(PAA)_4_·F_4_TCNQ						This work
	1.341	1.445	1.381	1.443	—	
(TOTA)_2_·F_4_BQ						This work
TOTA	1.402	1.390	1.389	1.386	1.393	
F_4_BQ	1.336	1.472	1.219	—	—	
TTF-F_4_BQ	1.328	1.470	1.212	—	—	44
2 TMIQ-F_4_BQ	1.316	1.470	1.210	—	—	45

a Average values from different structures in the provided references. For F_4_TCNQ^−^, the data for the various crystallized NBu_4_^+^ salts and for TOTA^+^ the data for the PF_6_^−^, ClO_4_^−^ and ReO_4_^−^ salts were used.

b Average values for two crystallographically different donor and acceptor molecules in the unit cell.

Table 2 summarizes pertinent bond lengths of reference compounds F_4_TCNQ, F_4_TCNQ^−^, TOTA, TOTA^+^, F_4_BQ, Cl_4_BQ, Cl_4_BQ^−^ and the D–A compounds of the present study. As can be seen from the data in Table 2, the metrics of the TOTA and the F_4_TCNQ constituents in (TOTA)_2_·(F_4_TCNQ)_2_·CH_2_Cl_2_ agree with those of the TOTA^+^ cation in the PF_6_^−^, ClO_4_^−^ and ReO_4_^−^ salts and of the F_4_TCNQ^−^ anion in NBu_4_^+^ F_4_TCNQ^−^, respectively.^37,38^ In particular, the TOTA constituent has completely flattened out (Fig. 5c). This characterizes (TOTA)_2_·(F_4_TCNQ)_2_·CH_2_Cl_2_ as a true CT salt with full ionicity.

**Fig. 6:**
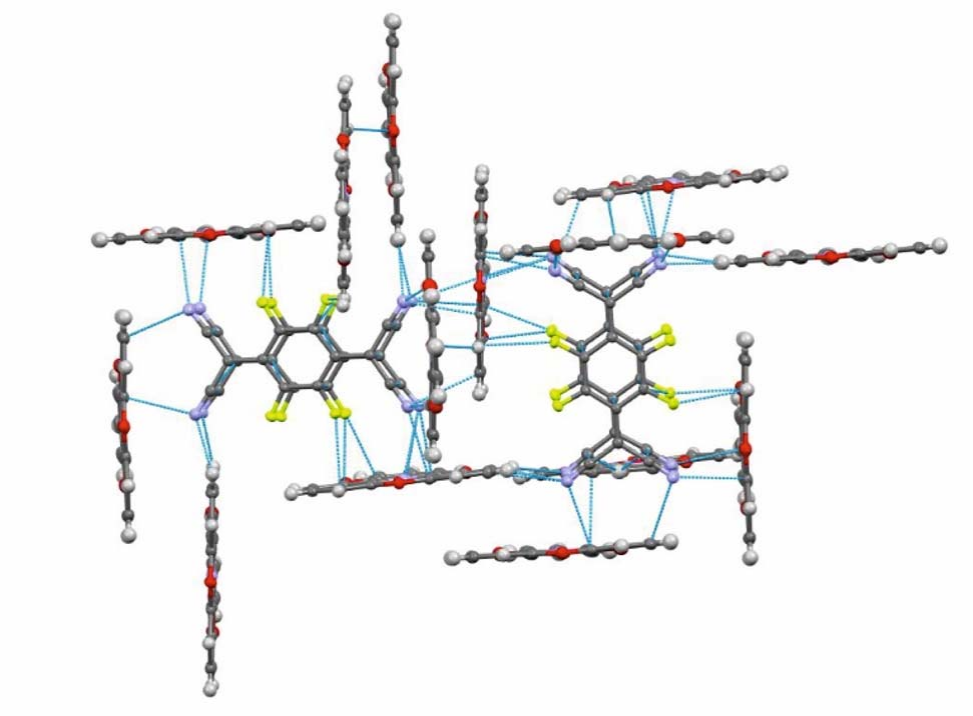
Intermolecular π-stacking, C≡N⋯ and C–F⋯π-hole tetrel and H-bonding interactions in (TOTA)_2_·(F_4_TCNQ)_2_·CH_2_Cl_2_, distances shorter than the sum of the van der Waals radii are indicated by blue dotted lines.

In the crystal lattice, the F_4_TCNQ^−^ anions associate to pairs of nearly parallel, ecliptically arranged molecules with a tilt angle of 1.60° between their ring planes and a rather small interplanar distance of 3.215 Å (Fig. 4 and 6). The formation of F_*n*_TCNQ^−^ (*n* = 0, 4) dimers has been observed on previous occasions and is associated with an antiferromagnetic alignment of their unpaired spins.^38,49–51^ Individual (F_4_TCNQ^−^)_2_ dimers are separated by two CH_2_Cl_2_ solvent molecules and arrange in columns that run parallel to the *a* axis of the unit cell. F_4_TCNQ^−^ ions of neighbouring columns are nearly coplanar with a modest tilt of their ring planes by 6.7° and rotated by almost 90°.

The F_4_TCNQ^−^ columns are separated by sheets that are formed by surrounding TOTA^+^ cations. Like the F_4_TCNQ^−^ anions, TOTA^+^ polycycles that belong to different sheets adopt nearly orthogonal orientations with interplanar angles of 85.8° and 88.0° between their ring planes. As is shown in Fig. 4b, the F_4_TCNQ^−^ dimers are encaged by six TOTA^+^ cations and every TOTA cation is in turn surrounded by three F_4_TCNQ^−^ dimers. This rather curious packing arrangement is established by various C≡N⋯ and C–F⋯π-hole tetrel bonds^26–30^ as well as by C–H⋯N≡C hydrogen bonds and one weak CH⋯F interaction of 2.640 Å. Fig. 6 provides a view of two neighbouring (F_4_TCNQ^−^)_2_(TOTA^+^)_6_ cages with two additional weakly associated TOTA^+^ cations and the ensuing network of noncovalent interactions. The C≡N⋯π-hole tetrel interactions range from 2.998 Å to 3.183 Å, while the C–F⋯π-hole contacts measure 2.977 Å to 3.136 Å; CH⋯N interactions cover a range from 2.500 Å to 2.701 Å. Adjacent cages weakly associate by pairwise CH⋯O contacts of 2.697 Å between parallel displaced and laterally offset TOTA^+^ cations. When viewed along the *b*-axis of the unit cell (see the horizontal rows in Fig. 4b), an alternating arrangement of (F_4_TCNQ^−^)_2_ π-dimers and two coplanar TOTA^+^ cations emerges. The D^+^⋯D^+^ π–π interactions of 3.388–3.445 Å are notably weaker than the A^−^⋯A^−^ π–π interactions of 3.108–3.195 Å (Fig. 4 and 6).

**Fig. 7:**
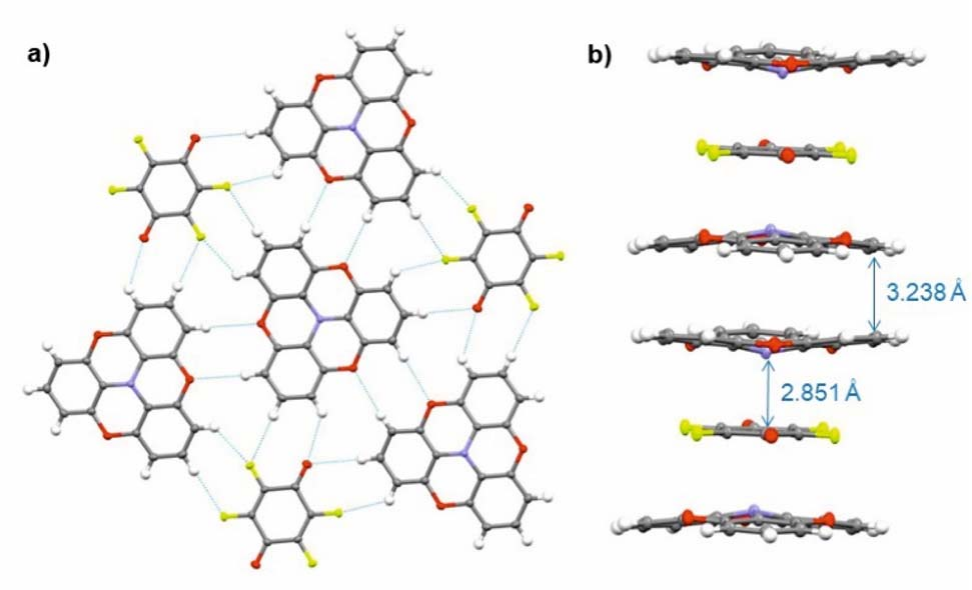
X-ray molecular structure of (TOTA)_2_ F_4_BQ. (a) Packing diagram within the *ab* plane with intermolecular H-bonding interactions indicated by blue dotted lines. (b) One-dimensional [D⋯A⋯D]_∞_ columns that run along the *c* axis of the unit cell. Colour codes: H, white; C, grey; N, blue; F, yellow-green; O, red.

Single crystals of (TOTA)_2_·F_4_BQ were grown by slow evaporation of a CH_2_Cl_2_ solution of their equimolar mixture. The asymmetric unit cell contains one TOTA donor molecule and half a F_4_BQ acceptor molecule. In the crystal lattice, each F_4_BQ acceptor molecule is surrounded by two slightly bowl-shaped TOTA donors to provide a centrosymmetric arrangement D⋯A⋯D of nearly coplanar TOTA and F_4_BQ molecules with interplanar angles of 3.6° between their planes as defined by the three oxygen atoms at the TOTA ether straps or the central C_6_ ring of F_4_BQ. The N atom of the TOTA donor is displaced by 0.25 Å from the TOTA ring plane and points towards the F_4_BQ acceptor to provide a N⋯F_4_BQ_centr._ distance of 2.851 Å. These D⋯A⋯D arrays stack into infinite columns that run along the *c*-axis of the unit cell. Fig. 7b provides a view of two such D⋯A⋯D triples; an extended view over several unit cells down the *c* axis can be found as Fig. S5 of the ESI.† Neighbouring donors have an interplanar distance of 3.791 Å between the centroids as defined by their oxygen atoms and form pairwise contacts C5⋯C15 of 3.238 Å. Within the *ab*-plane of the unit cell, every F_4_BQ acceptor associates with six coplanarly arranged TOTA donors through a total of eight C–F⋯H–C hydrogen bonds of 2.485 Å to 2.643 Å and four O⋯H–C hydrogen bonds of 2.567 Å and 2.614 Å, respectively (see Fig. S6 of the ESI†). The TOTA molecules that surround the F_4_BQ acceptors connect through pairwise O⋯H–C hydrogen bonds of 2.656 Å, respectively. In turn, every TOTA molecule is surrounded by alternately arranged TOTA donors and F_4_BQ acceptors. Fig. 7a provides a view of the molecule arrangement and the resulting H-bonding network.

The bonding parameters of both, the F_4_BQ acceptor and the TOTA donor argue for an only limited degree of charge transfer, but do not allow for a quantitative assessment. Since no X-ray data for an authenticated F_4_BQ^−^ anion seem to be available in the literature, we resort to its chloro-substituted analogue Cl_4_BQ^*n*−^ (*n* = 0, 1) for comparison.^43^ One-electron reduction of Cl_4_BQ causes a lengthening of the intracycle C=C bonds and, by a larger margin, the external C=O bonds while the former C–C bonds contract. The bond parameters of the F_4_BQ constituent of (TOTA)_2_·F_4_BQ are close to that of F_4_BQ itself or to those of its 1 : 1 TTF or its 1 : 2 TMIQ CT compounds, which also show only a fair degree of CT (TMIQ represents the 1,4-phenylene-bridged ditopic bis-carbazole donor shown on the right of the header of Table 2).^44,45^ In further agreement with an only modest degree of CT, the donor constituents retain the domed, non-planar structure of neutral TOTA, albeit with a smaller cone angle of 7.5° at the N atom and slightly wider C_ph_–N–C_ph_ bond angles *α*, *β*, and *γ* of 116.57(10)°, 116.86(10)° and 117.00(10)° as compared to the values of 115.3(2)°, 115.6(2)° and 115.7(2)° for neutral TOTA (Fig. 5).^31^ As already mentioned, TOTA^+^ is planar with angles *α*, *β*, and *γ* close to 120° (*e.g.* 119.7(5)°, 119.9(5)° and 120.3(5)° in the perrhenate salt).^27^ The N–C_phenyl_ and O–C_phenyl_ bond lengths in (TOTA)_2_·F_4_BQ are nearly identical to those of pristine, neutral TOTA and significantly longer than in (TOTA)_2_·(F_4_TCNQ)_2_·CH_2_Cl_2_ or other salts with authenticated TOTA^+^ cations.^31,32^

### The donor–acceptor compound (PAA)_4_·F_4_TCNQ

**Fig. 8:**
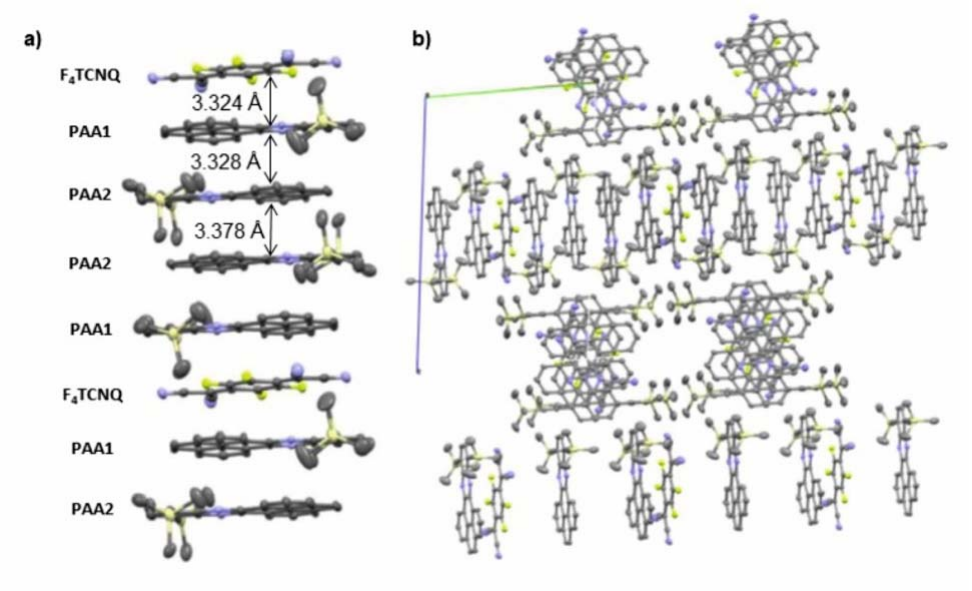
(a) Molecular packing of (PAA)_4_·F_4_TCNQ viewed along the *a* axis of the unit cell. (b) Alignment of the rows in the crystal lattice. Solvent molecules are removed for clarity reasons.

As suggested by the high anodic peak potential of 1205 mV, the pyrene-annulated azaacene PAA (Fig. 3) is only a very poor donor. Hence, the formation of a binary D–A compound was only observed with the strongest acceptor F_4_TCNQ. The dark green crystals obtained by slow evaporation of CH_2_Cl_2_ from a 1 : 1 solution of PAA and F_4_TCNQ turned out to assume a rather unusual 4 : 1 D : A stoichiometry (PAA)_4_·F_4_TCNQ. The compound crystallized in the monoclinic space group *C*2/*c*. The asymmetric unit consists of two molecules of the PAA donor and half a molecule of the F_4_TCNQ acceptor. In the crystal lattice, repeat units of four D and one A molecules form one-dimensional infinite columns [⋯D⋯D⋯A⋯D⋯D⋯]_∞_ that run along alternately the *a* or the *b* axis of the unit cell (Fig. 8). The neighbouring, crystallographically unique PAA donors PAA1 and PAA2 of an A⋯D⋯D⋯ sequence align in a quasi-centrosymmetric fashion in order to minimize steric repulsion between the bulky trimethylsilyl substituents. As is shown in the left panel of Fig. 8, these building blocks repeat in a centrosymmetric manner to generate columns. With interplane angles F_4_TCNQ–PAA1 of 2.30° and PAA1–PAA2 of 1.96°, the individual donor and the acceptor molecules arrange in a nearly coplanar fashion. Separations of 3.324 Å (F_4_TCNQ–PAA1), 3.328 Å (PAA1–PAA2) and 3.378 Å (PAA2–PAA2) are all smaller than 3.5 Å, which indicates π-stacking interactions.^52^

The close match between the bond lengths of the A constituent of this compound to those of neutral F_4_TCNQ evidences the lack of any substantial degree of CT from PAA. The bonding parameters of the crystallographically distinct PAA donors in (PAA)_4_·F_4_TCNQ differ slightly (see Fig. S7 of the ESI†), but are in line with those in neutral, pyrene-appended azaacene derivatives and other bis(trialkylsilyl) derivatives of diethynyl-substituted azaacenes.^23,24^

As a bottom line, crystallographic data indicate full CT in (TOTA)_2_·(F_4_TCNQ)_2_, an only moderate degree of CT in (TOTA)_2_·F_4_BQ, and the absence of any significant degree of CT in (PAA)_4_·F_4_TCNQ, which complies with the difference of oxidation potentials of the respective donor and the reduction potential of the acceptor.

### IR, UV-vis-NIR and EPR spectroscopy

In addition to structure data, infrared (IR) and UV-vis-NIR spectroscopy have proven highly instructive for quantifying the extent of charge-transfer in D–A compounds. Of particular diagnostic value are the redox state-dependent band positions of the nitrile C≡N *ν̃* (CN) or the quinone C=O stretching modes *ν̃* (CO) of the acceptor. Their shift with respect to the neutral and the reduced forms of the respective acceptor scales linearly with the fraction of charge *ρ* (in units of the elementary charge *e*) transferred from the donor to the acceptor.^53,54^ These band shifts provide a widely applied criterion for assessing the degree of CT.^6,21,55–60^ Arene stretching and bending vibrations of the D component may provide additional information.

**Fig. 9:**
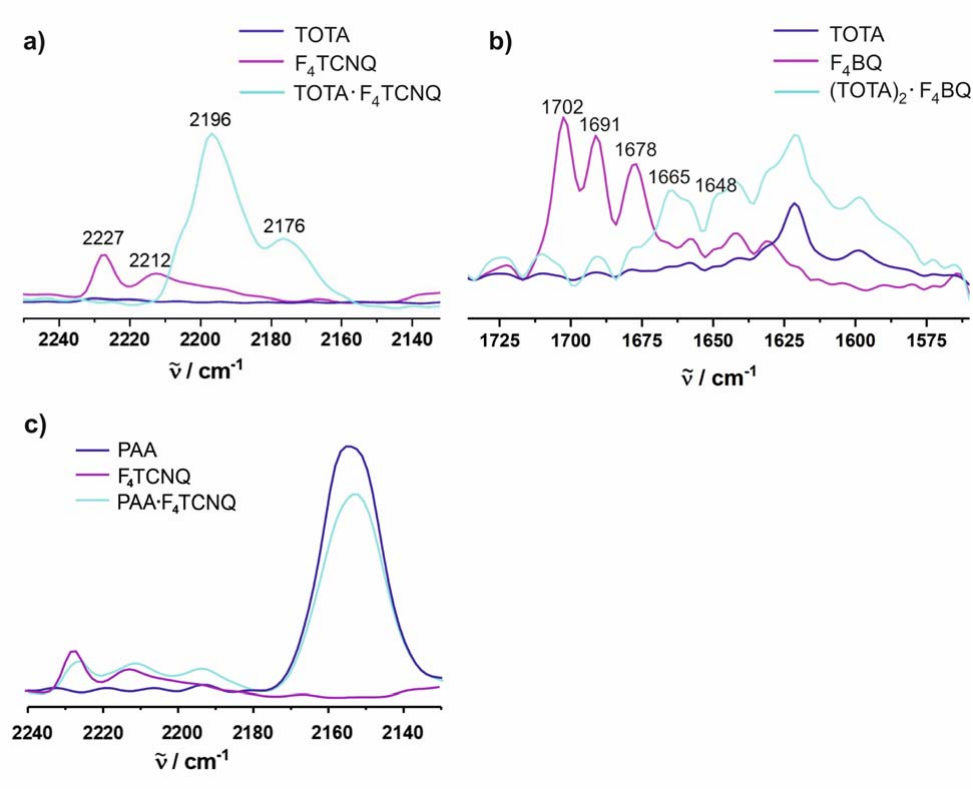
Spectroscopic changes in the mid IR region (C≡N stretching vibrations) of F_4_TCNQ in (a) (TOTA)_2_·(F_4_TCNQ)_2_·CH_2_Cl_2_, (b) (TOTA)_2_·F_4_BQ, and (c) (PAA)_4_·F_4_TCNQ.

The CN stretches of the F_4_TCNQ acceptor in TOTA·F_4_TCNQ at *ν̃*= 2196 cm^−1^ and 2176 cm^−1^ are significantly red-shifted from their positions of 2227 cm^−1^ (the stronger *b*_1u_ mode) and 2212 cm^−1^ (the weaker *b*_2u_ mode) in pristine F_4_TCNQ (Fig. 9a)^61^ and closely resemble those of the F_4_TCNQ^−^ anion of 2194 cm^−1^ and 2172 cm^−1^.^6,61,62^ The degree of charge-transfer *ρ* to F_4_TCNQ can be calculated according to eqn (1),

1 *ρ* = (2·Δ*ν̃*/*ν̃*_0_)·(1−*ν̃*_−1_^2^/*ν̃*_0_^2^)^−1^ (ref. **6 and 54**)

where *ν̃*_0_ and *ν̃*_-1_ represent the wavenumber of the more prominent C≡N stretching vibration in neutral F_4_TCNQ (*ν̃*_0_) or its associated radical anion (*ν̃*_-1_), and Δ*ν̃* is the difference between the band position in neutral F_4_TCNQ and the charge transfer compound, *ν̃*_0_ − *ν̃*_CT_, respectively. Applying eqn (1) to TOTA·F_4_TCNQ yields *ρ* = 0.95 in agreement with essentially full electron transfer. IR data for the D constituent agree with this view. Hence, IR(KBr) spectra of TOTA·F_4_TCNQ feature donor bands at 1590, 1334, 1277, 1074 and 1031 cm^−1^ (see Fig. S8 of the ESI†). These values are nearly identical to those of 1589, 1333, 1275, 1072 and 1028 cm^−1^ reported in the literature for the TOTA^+^ radical cation and differ from those of pristine TOTA (1621, 1480, 1336, 1318, 1265, 1067 and 1018 cm^−1^).^63^

**Fig. 10:**
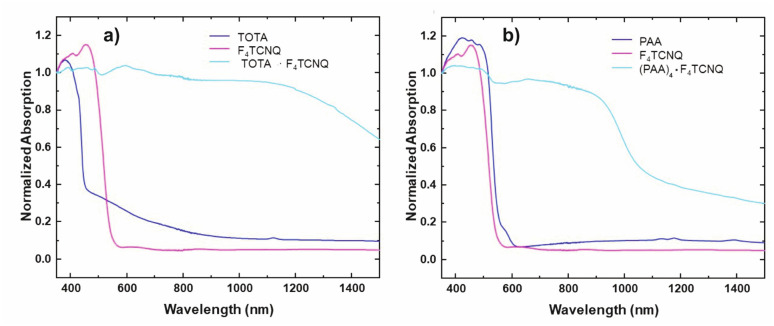
Monitoring the CT compound formation by UV-vis-NIR spectroscopy. The spectra of CT compounds are plotted with their parent donor and acceptor constituents. (a) (TOTA)_2_·(F_4_TCNQ)_2_·CH_2_Cl_2_ and (b) (PAA)_4_(F_4_TCNQ).

The formation of D–A compounds is also evident from UV-vis-NIR spectroscopy. UV-vis-NIR spectra of solid samples of neutral TOTA and F_4_TCNQ and of the CT compound TOTA·F_4_TCNQ were recorded in an integrating sphere in order to diminish intensity losses due to scattering and reflection. As shown in Fig. 10, the spectra of the neutral precursors display intense bands at 380 and 525 nm and 405 and 455 nm, respectively, whereas TOTA·F_4_TCNQ shows discernible peaks at 595, 650, 770 and at *ca.* 1060 nm.

**Fig. 11:**
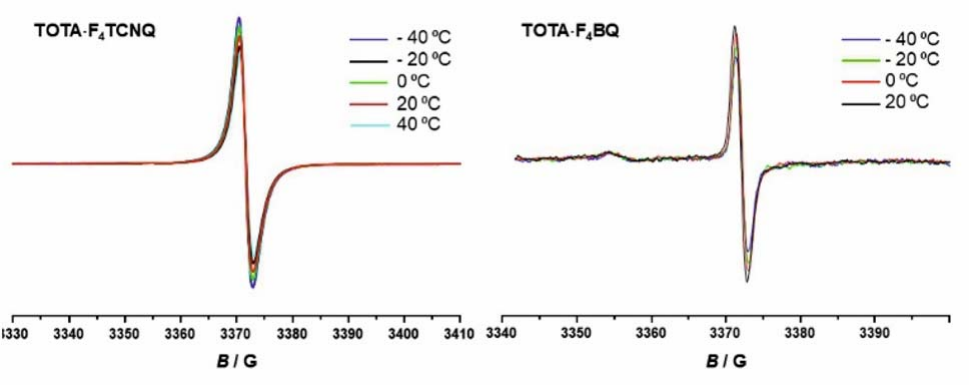
*T*-dependent EPR spectra of the CT compounds TOTA·F_4_TCNQ (left) and (TOTA)_2_·F_4_BQ (right).

EPR spectroscopy provides a highly sensitive probe of paramagnetic species resulting from charge transfer.^62,64,65^ Solid TOTA·F_4_TCNQ (Fig. 11a) shows accordingly an intense EPR resonance at a *g* value of 1.9990, whose intensity increases on cooling. This agrees with the *T*-dependent Boltzmann distribution as given in eqn (2)^66,67^ and the high ionicity in the ground state of this compound. One should note here that the observed EPR resonance in the solid state is likely due to exclusively the TOTA^+^ cation, as the F_4_TCNQ^−^ anions associate to diamagnetic dimers.2
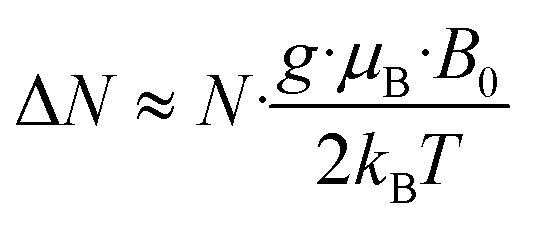


For F_4_BQ, the energies of the *ν*(CO) stretching vibrations serve the same purpose as the CN bands in X_*n*_TCNQ derivatives (X = Hal, *n* = 0–4) such that eqn (1) applies accordingly. *ν*_CO_ bands of F_4_BQ in (TOTA)_2_·F_4_BQ are found at 1665 and 1648 cm^−1^, whereas they are located at 1702, 1691 and 1678 cm^−1^ in neutral F_4_BQ (see Fig. 9b). The data are to be compared with literature values of 1705, 1693 and 1686 cm^−1^ for F_4_BQ^68^ and 1556 and 1502 cm^−1^ for the sodium salt of F_4_BQ^−^.^69,70^ Using eqn (1), *ρ* was calculated as 0.26. Hence, (TOTA)_2_·F_4_BQ seems to exhibit a fair degree of CT which was not so evident from the structure data. There are only minor shifts in the IR bands of the TOTA constituents (Fig. S9 of the ESI†). One should, however, note that the extent of charge loss from an individual TOTA donor is only half of that which is accumulated at the F_4_BQ acceptor. In the solid state, (TOTA)_2_·F_4_BQ exhibits a weak, broad CT absorption at low energy (Fig. S10 of the ESI†). As shown in Fig. 11b, solid TOTA·F_4_BQ is also EPR active, but shows an opposite *T* dependence to TOTA·F_4_TCNQ, i. e. the signal intensity decreases on lowering the temperature. This suggests that CT in these compounds is a thermally activated process. In summary, IR, UV-vis-NIR and EPR data on the solid samples are consistent with the notion of (nearly) quantitative CT from the donor to the acceptor in TOTA·F_4_TCNQ and a more modest one in (TOTA)_2_·F_4_BQ. The degree of CT *ρ* as quantified by the shift of the C≡N stretching vibrations of the donor amounts to 0.95 and 0.26, respectively.

In contrast, the IR data of (PAA)_4_(F_4_TCNQ) resemble those of the pristine, neutral constituents closely with only a small shift of the *ν*_CN_ band by *ca.* 1.5 cm^−1^ (Fig. 9c) which translates into *ρ* ≈ 0.05, thus indicating a very modest degree of CT (see also Fig. S11 of the ESI† for the arene bands). In agreement with the small degree of CT, (PAA)_4_(F_4_TCNQ) shows only a very weak EPR resonance signal (see Fig. S12 of the ESI†). Nevertheless, the compound absorbs strongly in the solid state over the entire UV and vis range down to 1100 nm as shown in Fig. 10b. We note that accounts of compounds showing prominent CT bands despite small degrees of CT have appeared in the literature.^19^

In order to assess their conductive properties, single crystals of all three isolated CT compounds were placed on a gold plate or a conductive Cu-tape and contacted with two closely spaced nanoprobes, which served as electrodes (for details to the experimental setup, see the Materials and methods section and Fig. S13 of the ESI†). Even on applying a maximum voltage of 20 V, no detectable current flow was observed for TOTA·F_4_TCNQ and for (PAA)_4_·F_4_TCNQ, even at a tip distance as small as *ca.* 10 μm. Their insulating behaviour (Fig. S14 and S15 of the ESI†) is a direct consequence of their structures with very weakly interacting (TOTA^+^)_8_(F_4_TCNQ^−^)_2_ cages or [⋯D⋯D⋯A⋯D⋯D⋯]_∞_ columns with negligible charge transfer. (TOTA)_2_·F_4_BQ is also nearly insulating with a resistance per unit length of 5–10 GΩ μm^−1^ for different specimen, which is close to the detection limit of our experimental setup (Fig. S16–S19 of the ESI†).

## Conclusions

We describe two charge-transfer compounds of the 2,2′ : 6′,2′′ : 6′′,6-trioxotriphenylamine donor and tetrafluoro-tetracyano-*p*-quinodimethane (F_4_TCNQ) or tetrafluoro-*p*-benzoquinone (F_4_BQ) as the acceptor component and a donor–acceptor complex of a much less electron-rich pyrene-annulated azaacene (PAA) with F_4_TCNQ. Although in each case equimolar amounts of the donor D and the acceptor A were used for their synthesis, only (TOTA)_2_·(F_4_TCNQ)_2_·CH_2_Cl_2_ adopted a D : A stoichiometry of 1 : 1. Crystals isolated with F_4_BQ as the acceptor provided a 2 : 1 ratio between the donor and the acceptor constituents instead. The combination of PAA and F_4_TCNQ resulted even in a rare 4 : 1 composition, yielding (PAA)_4_·(F_4_TCNQ). X-ray diffraction analysis indicated full charge transfer from the donor to the acceptor in (TOTA)_2_·(F_4_TCNQ)_2_·CH_2_Cl_2_ by virtue of the bond parameters of the F_4_TCNQ acceptor and the planarization of the TOTA constituent. These findings are also supported by IR and UV-vis-NIR spectroscopy. In the crystalline state, π-stacked F_4_TCNQ^−^ dimers are encaged by six TOTA^+^ counterions with only weak interactions between these structural entities.

For the other two D/A combinations, X-ray structure analysis revealed mixed stacking patterns with [D⋯A⋯D]_∞_ or [D⋯D⋯A⋯D⋯D]_∞_ packing motifs. Monitoring the shifts of the C≡N and C=O stretching frequencies in the CT compounds with respect to the neutral and monoreduced acceptors revealed an essentially complete charge-transfer in (TOTA)_2_·(F_4_TCNQ)_2_·CH_2_Cl_2_, a moderate degree of charge-transfer in (TOTA)_2_·(F_4_BQ) (*ρ* = 0.26) and an only very modest degree of CT in (PAA)_4_·(F_4_TCNQ). Nevertheless, a fairly intense CT band was found in solid state UV-vis-NIR spectra. *T*-dependent EPR spectra recorded in the solid state agree with a (nearly) complete CT in the ground state of the TOTA·F_4_TCNQ compound, while CT in (TOTA)_2_·(F_4_BQ) is thermally activated and (PAA)_4_·(F_4_TCNQ) shows only a very weak EPR signal. All CT compounds are non-conductive or, in the case of (TOTA)_2_·(F_4_BQ) very weakly conductive in the solid state.

## Data availability

Details to the methods and materials, characterization data and additional figures can be found in the ESI†. CCDC reference numbers for the reported crystallographic structures are 2220394 ((PAA)_4_·F_4_TCNQ), 2220397 ((TOTA)_2_·F_4_BQ) and 2220398 ((TOTA)_2_·(F_4_TCNQ)_2_·CH_2_Cl_2_).

## Conflicts of interest

There are no conflicts to declare.

## Supplementary Material

RA-013-D2RA07322F-s001

RA-013-D2RA07322F-s002
