# Supplementary material for: Organic binary charge-transfer compounds of 2,2′ : 6′,2′′ : 6′′,6-trioxotriphenylamine and a pyrene-annulated azaacene as donors
Source: RSC Adv. 2023 Jan 30;13(6):3652–60. doi: 10.1039/d2ra07322f (PMC9890512; doi:10.1039/d2ra07322f)
Supplement: RA-013-D2RA07322F-s001 [file RA-013-D2RA07322F-s001.pdf]

Electronic Supplementary Material (ESI) for RSC Advances.
This journal is © The Royal Society of Chemistry 2023

Organic binary charge-transfer compounds of 2,2′:6′,2′′:6′′,6-trioxotriphenylamine and a pyrene-annulated azaacene as donors

Rajorshi Das,[a] Michael Linseis,[a] Stefan M. Schupp,[b] Franciska S. Gogesch,[a] Lukas Schmidt-Mende,[b] and Rainer F. Winter *[a]

[a]*Fachbereich Chemie, Universität Konstanz, Universitätsstrasse 10, 78457 Konstanz (Germany)*

[b]*S. M. Schupp, Prof. Dr. L. Schmidt-Mende, Fachbereich Physik, Universität Konstanz, Universitätsstrasse 10, 78457 Konstanz (Germany)*

To whom correspondence should be addressed: E-mail: rainer.winter@uni-konstanz.de.

Materials and methods

General Methods. All manipulations were performed under an atmosphere of purified nitrogen with dry, distilled, and nitrogen-saturated solvents. Donors 2,2′:6′,2″:6″,6-trioxotriphenylamine (TOTA)[1] and pyrene-annulated azaacene (PAA)[2] were synthesized according to the previously reported procedures. All acceptors F_4TCNQ, TCNQ, F_4BQ, Cl_4BQ and Br_4BQ were purchased from commercial sources and were further purified via sublimation before use.

IR-, UV-vis-NIR and EPR Spectra Measurements. FT-IR spectra were recorded on a Bruker TensorIII instrument. UV-vis-NIR spectra on solid samples were obtained on a Cary 5000 UV-Vis-NIR spectrometer. The powderous sample was pressed between two glass slides, put into a sample holder and inserted into the centre position of an integrating sphere, which diminishes scattering and reflection losses. Electron paramagnetic resonance (EPR) studies were performed on a table-top X-band spectrometer MiniScope MS 400 from magnettec. Simulation of the experimental EPR spectra was performed with the MATLAB EasySpin program.

X-Ray Crystallography. X-Ray diffraction analysis was performed on a STOE IPDS-II diffractometer (STOE & CIE GmbH, Darmstadt, Germany) equipped with a graphite-monochromated MoK_α radiation source (λ = 0.71073 Å) or, in the case of $(PAA)_4 \cdot F_4TCNQ$, a CuK_α radiation source (λ = 1.54186 Å) and an image plate detection system at T = 100.15 K. Using *Olex2*,[3] the structures were solved with the *ShelXT*[4] structure solution program using Intrinsic Phasing and refined with the *ShelXL*[4] refinement package using Least Squares minimization. Hydrogen atoms were introduced at their calculated positions. Structure plots were generated with the Mercury program.[5] Crystallographic data were deposited at the Cambridge Crystallographic Data Centre and can be retrieved with their CCDC reference numbers 2220394 ($(PAA)_4 \cdot F_4TCNQ$), 2220397 ($(TOTA)_2 \cdot F_4BQ$) and 2220398 ($(TOTA)_2 \cdot (F_4TCNQ)_2 \cdot CH_2Cl_2$).

Conductivity Measurements. Conductivity measurements were performed with single crystals of charge-transfer compounds. Crystals were placed on a gold plate or a conductive Cu-tape, which was kept on a scanning electron microscope (SEM) stamp. Micrometer-sized tungsten probe tips were used as electrodes. During measurements, the applied voltage was varied from -20 to 20 V.

Experimental procedures

$(TOTA)_2\cdot(F_4TCNQ)_2\cdot CH_2Cl_2$. 2,2′:6′,2″:6″,6-Trioxotriphenylamine (TOTA, 1.4 mg, 0.005 mmol) and 2,3,5,6-tetrafluoro-7,7,8,8-tetracyanoquinodimethane (F_4TCNQ, 1.4 mg, 0.005 mmol) were dissolved in dichloromethane (4 mL) via sonication. The solvent was allowed to slowly evaporate. After 3 days, dark purple crystals of $(TOTA)_2\cdot(F_4TCNQ)_2\cdot CH_2Cl_2$ were obtained.

Yield: Quantitative.

IR (KBr, cm^{-1}): 2196 (s), 2176 (s), 1641 (m), 1631 (s), 1613 (s), 1590 (s), 1502 (s), 1482 (m), 1385 (s), 1277 (m), 1200 (w), 1142 (w), 1075 (m), 1030 (m).

EPR: g_{iso}-value = 1.9990

Elemental (CHN) Analysis: C: 60.80, H: 2.04, N: 11.50 (Calcd. C: 60.46, H: 1.66, N: 11.56 for $(TOTA)_2\cdot(F_4TCNQ)_2\cdot CH_2Cl_2$ ($C_{61}H_{20}N_{10}O_6F_8Cl_2$).

$(TOTA)_2\cdot F_4BQ$. 2,2′:6′,2″:6″,6-Trioxotriphenylamine (TOTA, 1.4 mg, 0.005 mmol) and tetrafluoro-1,4-benzoquinone (F_4BQ, 0.9 mg, 0.005 mmol) were dissolved in dichloromethane (6 mL) via sonication. The solution was allowed to slowly evaporate. After 4 days, dark orange crystals of $(TOTA)_2\cdot F_4BQ$ were obtained, which were manually separated for further studies.

IR (KBr, cm^{-1}): 1664 (m), 1641 (m), 1620 (s), 1599 (m), 1526 (m), 1502 (m), 1482 (vs), 1402 (w), 1384 (w), 1357 (w), 1338 (s), 1318 (m), 1266 (s), 1157 (w), 1069 (s), 1017 (s).

EPR: g_{iso}-value = 1.9880

Elemental (CHN) Analysis: C: 67.04, H: 2.16, N: 3.75 (Calcd. C: 66.85, H: 2.40, N: 3.71 for $(TOTA)_2\cdot(F_4BQ)$, $C_{42}H_{18}F_4N_2O_4$).

Synthesis of $(PAA)_4\cdot F_4TCNQ$: Pyrene-annulated azaacene (PAA, 4.9 mg, 0.01 mmol) and 2,3,5,6-tetrafluoro-7,7,8,8-tetracyanoquinodimethane (F_4TCNQ, 2.8 mg, 0.01 mmol) were dissolved in dichloromethane (4 mL) via sonication. The solvent was allowed to slowly evaporate. After 3 days, dark red crystals of the title compound were obtained and collected.

IR (KBr, cm^{-1}): 2225 (s), 2212 (m), 1622 (m), 1607 (m), 1587 (s), 1565 (m), 1482 (s), 1459 (s), 1423 (w), 1402 (m), 1383 (s), 1352 (s), 1334 (s), 1308 (s), 1248 (vs), 1150 (s), 1112 (s), 1086 (m), 1042 (m).

EPR: g_{iso}-value = 1.9898.

Elemental (CHN) Analysis: C: 73.87, H: 4.81, N: 7.59 (Calcd. C: 74.30, H: 4.99, N: 7.43 for $(PAA)_4\cdot(F_4TCNQ)$, $C_{70}H_{56}F_2N_6Si_4$).

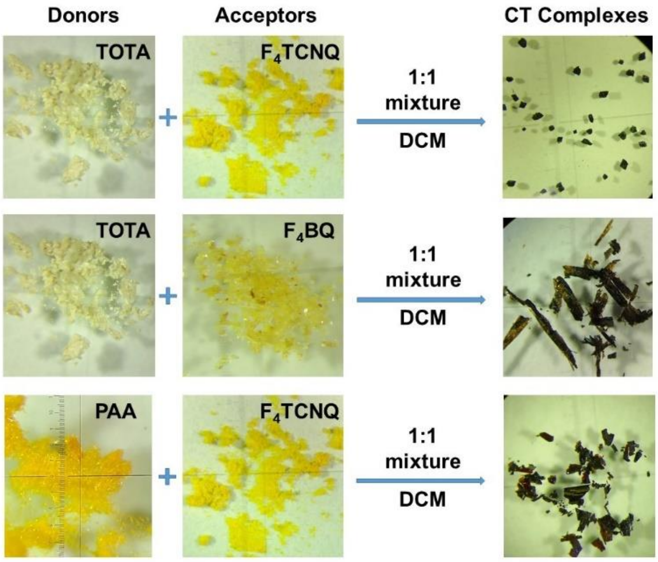

Figure S1. Photographs of the crystalline CT compounds of this study and their precursors.

Cyclic Voltammetry

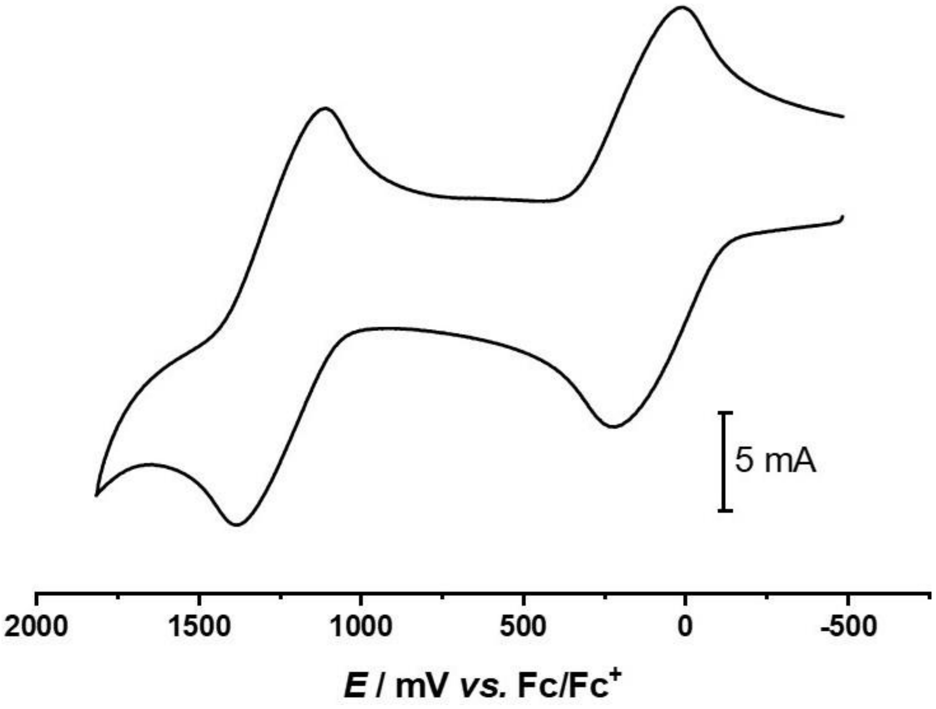

Figure S2. Cyclic voltammogram of **TOTA** at v = 100 mV/s with 0.1 M $TBAPF_6$ as the supporting electrolyte in dichloromethane at 295(±3) K.

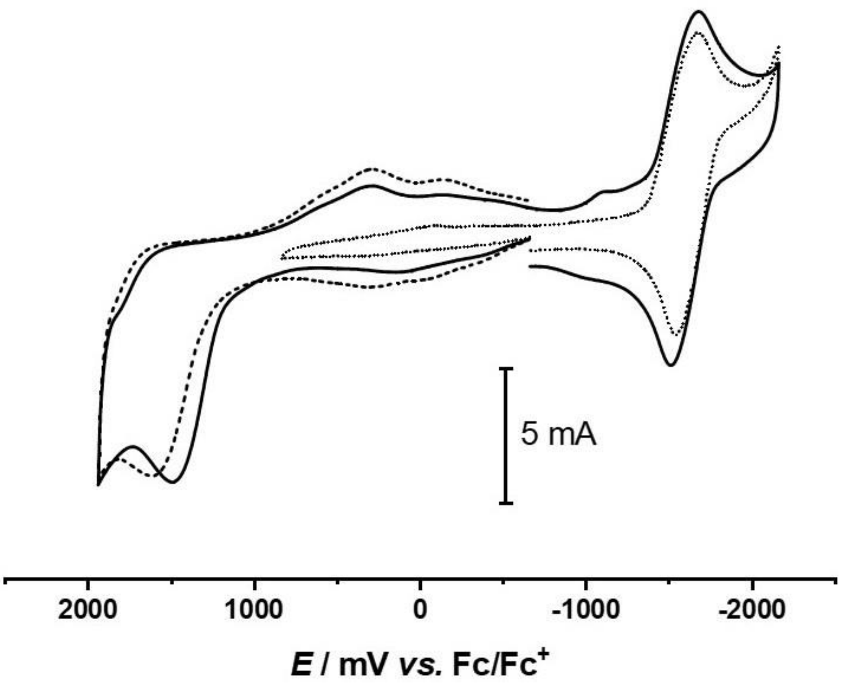

Figure S3. Cyclic voltammogram of **PAA** at *v* = 100 mV/s with 0.1 M $TBAPF_6$ as the supporting electrolyte in dichloromethane at 295(±3) K. The continuous line (▬) represents the full cyclic voltammogram with the oxidation and the reduction waves of PAA. The dashed line (---) represents only the oxidation of PAA. The dotted line (····) represents only the reduction of PAA.

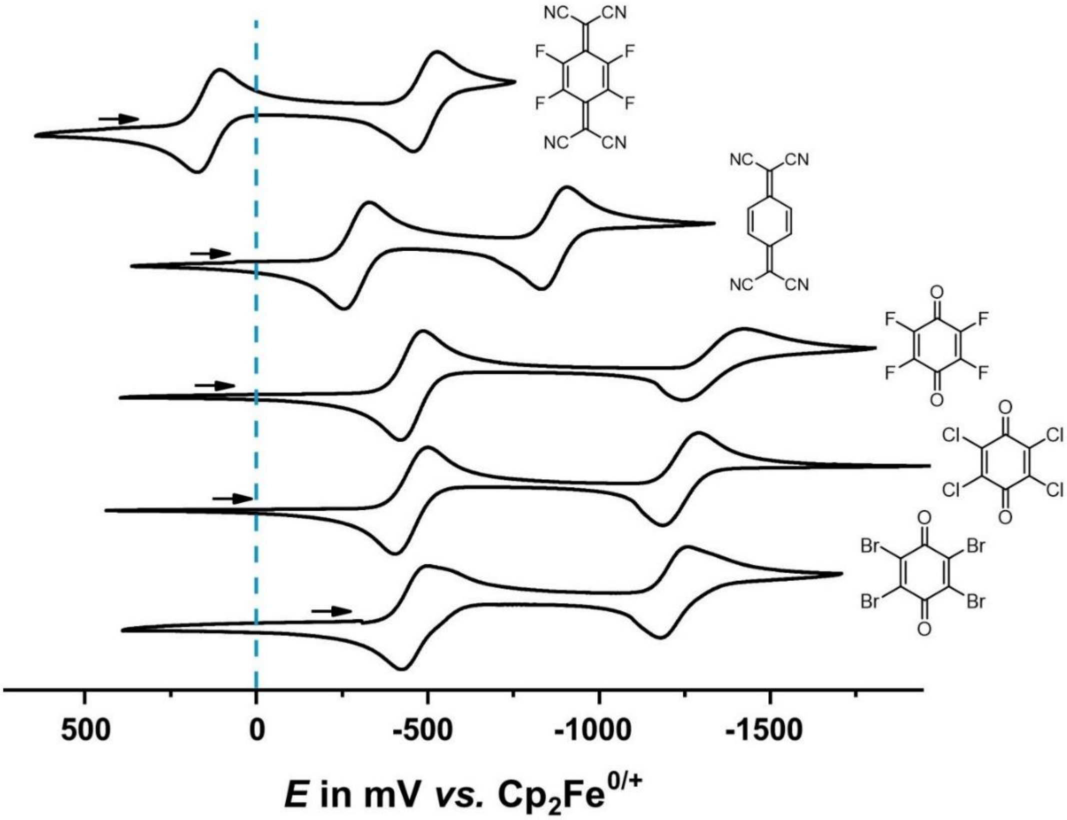

Figure S4. Cyclic voltammograms of all acceptors used in this study at *v* = 100 mV/s with 0.1 M $TBAPF_6$ as the supporting electrolyte in dichloromethane at 295(±3) K.

X-ray Diffraction Analysis

Table S1. Crystal data and structure refinement for $(TOTA)_2 \cdot (F_4TCNQ)_2 \cdot CH_2Cl_2$.

Empirical formula	$C_{61}H_{20}Cl_2F_8N_{10}O_6$
Formula weight	1211.77
Temperature/K	100
Crystal system	monoclinic
Space group	$P2_1/c$
a/Å	17.1173(6)
b/Å	19.0311(5)
c/Å	16.1480(6)
α/°	90
β/°	109.490(3)
γ/°	90
Volume/Å^3	4959.0(3)
Z	4
ρ_{calc} g/cm^3	1.623
μ/mm^{-1}	0.232
F(000)	2440.0
Crystal size/mm^3	0.5 × 0.3 × 0.2
Radiation	Mo Kα (λ = 0.71073)
2Θ range for data collection/°	3.688 to 55.38°
Index ranges	$-22 \leq h \leq 22$, $-24 \leq k \leq 24$, $-20 \leq l \leq 18$
Reflections collected	*29782*
Independent reflections	*11385 [R_{int} = 0.0283, R_{sigma} = 0.0279]*
Data/restraints/parameters	11385/0/785
Goodness-of-fit on F^2	1.050
Final R indexes [I ≥ 2σ (I)]	R_1 = 0.0488, wR_2 = 0.1047
Final R indexes [all data]	R_1 = 0.0804, wR_2 = 0.1292
Largest diff. peak/hole / e Å^{-3}	0.31/-0.45

Table S2. Fractional Atomic Coordinates (×10[4]) and Equivalent Isotropic Displacement Parameters (Å[2]×10[3]) for $(TOTA)_2\cdot(F_4TCNQ)_2\cdot CH_2Cl_2$. U_{eq} is defined as 1/3 of the trace of the orthogonalised U_{ij} tensor.

**Atom**	***x***	***y***	***z***	**U(eq)**
Cl2	4951.4(5)	732.7(6)	9046.4(5)	62.6(2)
Cl1	4717.1(5)	-735.3(6)	8476.1(6)	64.1(2)
F3	2669.8(8)	5284.8(7)	3709.5(8)	27.9(3)
F8	841.3(8)	6423.5(7)	4100.9(8)	28.4(3)
F2	3188.2(9)	3523.8(7)	5903.6(8)	30.0(3)
F6	1164.7(9)	3695.8(7)	5099.5(8)	29.8(3)
F4	2770.0(8)	6246.1(7)	4890.1(8)	28.3(3)
F1	3290.6(9)	4490.0(7)	7089.8(8)	29.8(3)
F7	734.9(8)	5463.2(7)	2920.7(8)	28.3(3)
F5	1253.5(9)	4656.6(7)	6280.6(8)	29.0(3)
O5	639.2(9)	1739.9(8)	5081.2(10)	23.8(3)
O6	3220.8(9)	2510.3(9)	7192.6(10)	26.7(3)
O4	3225.1(9)	804.0(8)	4984.9(10)	27.6(3)
O2	1226.9(9)	-1809.0(8)	3920.5(10)	26.5(3)
O3	3659.0(10)	-897.1(10)	6145.3(11)	36.2(4)
O1	3932.9(11)	-2683.2(10)	4143.8(12)	37.1(4)
N2	2361.3(11)	1687.8(10)	5747.0(11)	22.4(4)
N3	2826.5(12)	2487.9(10)	4350.2(12)	28.0(4)
N1	2936.3(11)	-1795.7(10)	4734.9(12)	25.9(4)
N4	2820.8(12)	4099.9(11)	2522.4(12)	28.9(4)
N10	1179.5(13)	5866.9(11)	7483.7(13)	31.1(4)
N8	813.8(13)	4236.2(11)	1715.6(13)	31.1(4)
N6	3237.4(13)	5772.3(11)	8310.9(13)	31.7(4)
N7	708.5(13)	2676.5(11)	3549.0(13)	32.6(5)
N5	3285.2(13)	7276.1(11)	6437.7(13)	32.1(4)
N9	1213.0(14)	7451.8(11)	5635.6(13)	36.0(5)
C37	3028.7(12)	5426.1(11)	6057.8(13)	21.1(4)
C26	2366.2(13)	2536.6(12)	6851.0(14)	23.2(4)
C19	1938.3(13)	1284.6(11)	5034.9(14)	22.5(4)
C43	2826.1(13)	3821.6(11)	4085.0(14)	22.7(4)
C20	2370.8(13)	848.6(12)	4642.4(14)	24.1(4)
C25	1934.7(13)	2124.5(12)	6125.8(14)	22.7(4)
C38	2885.5(13)	5572.1(11)	5159.7(14)	22.2(4)
C51	868.0(13)	5249.3(12)	3750.8(13)	23.0(4)
C56	844.5(13)	4148.6(12)	2430.5(14)	24.6(5)
C39	2833.8(13)	5070.7(12)	4544.7(13)	21.7(4)
C44	2819.9(13)	3989.4(11)	3222.8(14)	23.3(4)
C40	2907.7(12)	4346.3(11)	4732.9(13)	21.1(4)
C55	902.4(13)	3994.3(11)	3313.8(14)	22.6(4)
C49	1058.9(12)	5600.9(11)	5266.0(13)	21.0(4)
C46	3079.1(13)	5958.0(12)	6685.1(14)	23.3(4)
C58	1141.1(13)	6123.0(11)	5906.2(14)	22.4(4)
C32	3639.2(14)	1208.6(12)	5700.4(14)	25.5(5)
C24	1071.8(13)	1317.8(11)	4700.8(14)	23.3(4)
C30	1070.9(13)	2142.9(11)	5794.3(13)	22.3(4)

Table S2. Fractional Atomic Coordinates (×10^4) and Equivalent Isotropic Displacement Parameters ($Å^2$×10^3) for $(TOTA)_2$·$(F_4TCNQ)_2$·CH_2Cl_2. U_{eq} is defined as 1/3 of the trace of the orthogonalised U_{ij} tensor.

**Atom**	***x***	***y***	***z***	**U(eq)**
C54	1111.3(13)	4869.9(12)	5448.4(13)	22.0(4)
C45	2832.2(13)	3089.1(12)	4256.5(14)	22.9(4)
C59	1169.6(13)	5965.6(11)	6776.4(14)	23.8(4)
C29	637.0(14)	2561.8(12)	6180.1(14)	24.6(5)
C41	3064.7(13)	4196.5(11)	5635.1(14)	22.4(4)
C50	921.7(13)	5749.0(11)	4366.0(14)	21.6(4)
C31	3213.2(13)	1663.9(12)	6084.2(14)	24.1(4)
C52	939.3(12)	4518.6(11)	3939.1(13)	21.1(4)
C7	2445.4(14)	-1351.8(12)	5020.1(14)	24.7(5)
C23	645.5(14)	924.8(12)	3973.3(15)	26.2(5)
C36	3637.3(14)	2080.3(12)	6801.1(14)	26.1(5)
C8	1587.5(14)	-1359.6(12)	4605.9(14)	24.3(4)
C2	1729.0(14)	-2223.0(12)	3612.3(14)	25.9(5)
C47	3161.3(13)	5825.6(12)	7578.9(14)	25.1(5)
C42	3118.5(13)	4698.1(12)	6251.4(13)	21.8(4)
C60	1170.1(14)	6854.9(12)	5733.8(14)	25.4(5)
C48	3179.4(13)	6685.5(13)	6517.7(14)	25.3(5)
C53	1062.6(13)	4371.6(11)	4833.6(14)	22.1(4)
C1	2584.4(14)	-2227.1(12)	4025.1(14)	26.2(5)
C27	1940.1(14)	2963.7(12)	7235.7(14)	26.7(5)
C57	809.3(14)	3267.1(12)	3469.3(14)	25.2(5)
C21	1953.0(15)	455.0(12)	3920.8(15)	28.3(5)
C13	3785.4(14)	-1792.8(13)	5141.8(15)	30.2(5)
C3	1376.9(16)	-2634.9(12)	2883.4(15)	30.1(5)
C12	2806.2(14)	-906.6(13)	5735.0(15)	29.1(5)
C33	4491.2(14)	1172.0(14)	6043.1(15)	30.8(5)
C35	4492.6(14)	2058.2(14)	7134.2(16)	31.3(5)
C14	4273.9(15)	-2251.4(14)	4849.4(17)	34.3(6)
C6	3086.7(15)	-2662.1(13)	3719.5(16)	30.0(5)
C9	1093.4(15)	-918.3(12)	4888.8(15)	28.1(5)
C18	4136.3(15)	-1343.3(14)	5849.4(16)	33.3(5)
C28	1077.4(15)	2971.8(12)	6896.7(15)	27.1(5)
C10	1464.5(16)	-483.7(13)	5600.9(16)	31.4(5)
C22	1090.2(15)	499.2(12)	3599.2(15)	28.9(5)
C11	2319.7(16)	-469.7(13)	6032.6(16)	32.9(5)
C4	1887.4(17)	-3059.2(13)	2582.2(16)	35.2(6)
C34	4900.9(15)	1603.4(14)	6748.8(16)	34.0(6)
C5	2742.1(17)	-3078.1(13)	2997.6(16)	35.2(6)
C17	4987.5(16)	-1344.5(17)	6263.8(17)	43.5(7)
C15	5120.2(16)	-2264.5(17)	5272.8(18)	41.7(7)
C16	5462.3(16)	-1810.9(18)	5963.0(19)	46.8(7)
C61	4707.2(18)	149.0(19)	8147.0(19)	53.0(8)

Table S3. Anisotropic Displacement Parameters (Å^2×10^3) for $(TOTA)_2\cdot(F_4TCNQ)_2\cdot CH_2Cl_2$. The Anisotropic displacement factor exponent takes the form: $-2\pi^2[h^2a^{*2}U_{11}+2hka^*b^*U_{12}+\ldots]$.

**Atom**	**U_{11}**	**U_{22}**	**U_{33}**	**U_{23}**	**U_{13}**	**U_{12}**
Cl2	34.4(4)	102.1(7)	47.5(4)	-6.3(4)	8.5(3)	10.6(4)
Cl1	47.1(4)	88.9(7)	50.3(5)	11.6(4)	8.2(3)	-11.7(4)
F3	40.0(7)	26.0(7)	17.6(6)	2.5(5)	9.6(5)	2.2(6)
F8	39.1(7)	21.1(6)	23.8(6)	4.4(5)	8.9(6)	5.6(6)
F2	41.3(8)	22.1(7)	24.3(7)	5.0(5)	7.7(6)	4.2(6)
F6	45.3(8)	19.2(6)	24.5(7)	2.8(5)	11.3(6)	-0.9(6)
F4	41.4(8)	20.3(6)	22.8(6)	2.2(5)	10.2(6)	0.7(6)
F1	40.1(8)	30.3(7)	16.6(6)	4.0(5)	6.1(5)	1.5(6)
F7	36.9(7)	28.3(7)	16.5(6)	3.4(5)	4.9(5)	2.6(6)
F5	43.2(8)	26.4(7)	18.8(6)	2.5(5)	12.3(6)	-0.3(6)
O5	21.7(7)	27.2(8)	22.1(7)	-0.6(6)	6.8(6)	1.2(6)
O6	23.1(8)	32.7(9)	21.8(8)	-0.2(6)	4.2(6)	0.3(6)
O4	25.1(8)	30.5(9)	27.5(8)	-0.1(7)	9.2(6)	3.5(7)
O2	24.8(8)	30.1(9)	24.0(8)	1.2(6)	7.2(6)	-1.1(6)
O3	30.5(9)	44.3(11)	28.1(9)	-0.6(8)	2.3(7)	-5.4(8)
O1	33.4(9)	42.6(11)	38.4(10)	7.0(8)	16.2(8)	8.3(8)
N2	20.9(9)	26.1(10)	19.9(9)	2.4(7)	6.3(7)	2.1(7)
N3	30.5(10)	28.6(11)	23.8(9)	0.7(8)	7.5(8)	0.2(8)
N1	23.9(9)	30.4(10)	22.9(9)	5.7(8)	7.3(8)	1.5(8)
N4	32.5(10)	30.9(11)	23.9(10)	-2.8(8)	10.1(8)	0.6(8)
N10	41.4(12)	27.1(10)	27.4(10)	-0.8(8)	15.1(9)	-0.6(9)
N8	36.5(11)	31.0(11)	24.7(10)	-3.7(8)	8.9(8)	-4.1(9)
N6	38.2(11)	33.4(11)	24.1(10)	-2.5(8)	11.1(9)	-5.8(9)
N7	40.5(12)	30.7(12)	28.5(10)	-1.7(8)	14.3(9)	-3.4(9)
N5	38.8(11)	30.1(11)	27.7(10)	-2.3(8)	11.5(9)	-4.7(9)
N9	50.6(13)	28.0(11)	24.6(10)	1.6(8)	5.9(9)	-0.3(9)
C37	18.0(9)	25.0(11)	18.9(10)	-0.5(8)	4.2(8)	-1.8(8)
C26	24.6(10)	23.4(11)	19.8(10)	4.6(8)	5.2(8)	0.7(8)
C19	24.7(10)	23.3(11)	18.5(10)	2.2(8)	5.8(8)	0.4(8)
C43	21.3(10)	24.0(11)	21.9(10)	0.3(8)	6.0(8)	1.3(8)
C20	25.0(11)	23.6(11)	24.6(11)	4.1(9)	9.4(9)	2.1(9)
C25	23.6(10)	24.9(11)	19.9(10)	2.8(8)	7.9(8)	3.1(8)
C38	24.0(10)	20.3(10)	20.9(10)	1.9(8)	5.7(8)	-1.1(8)
C51	21.3(10)	28.3(11)	16.5(9)	4.2(8)	2.3(8)	1.0(9)
C56	24.6(11)	24.9(11)	22.2(11)	-4.3(9)	5.0(9)	-2.9(9)
C39	22.1(10)	26.3(11)	16.8(9)	2.8(8)	6.5(8)	-0.1(8)
C44	21.7(10)	21.9(11)	25.4(11)	-4.6(9)	6.6(9)	0.3(8)
C40	18.8(10)	24.3(11)	19.6(10)	1.6(8)	5.6(8)	-1.0(8)
C55	22.6(10)	24.1(11)	20.1(10)	1.2(8)	5.6(8)	-2.4(8)
C49	19.0(9)	23.1(11)	21.1(10)	0.5(8)	6.9(8)	0.3(8)
C46	23.6(10)	26.1(11)	19.6(10)	-0.5(8)	6.6(8)	-3.0(9)
C58	21.4(10)	23.4(11)	21.5(10)	1.5(8)	6.1(8)	2.7(8)
C32	26.4(11)	28.1(12)	22.3(10)	4.9(9)	8.4(9)	3.2(9)
C24	25.0(11)	23.2(11)	22.9(10)	2.3(8)	9.8(9)	2.9(8)
C30	25.5(11)	22.4(11)	18.3(10)	3.8(8)	6.2(8)	-0.6(8)
C54	23.8(10)	25.9(11)	15.1(9)	1.9(8)	4.9(8)	-0.7(8)

Table S3. Anisotropic Displacement Parameters ($Å^2×10^3$) for $(TOTA)_2·(F_4TCNQ)_2·CH_2Cl_2$. The Anisotropic displacement factor exponent takes the form: $-2\pi^2[h^2a^{*2}U_{11}+2hka^*b^*U_{12}+…]$.

Atom	U_{11}	U_{22}	U_{33}	U_{23}	U_{13}	U_{12}
C45	21.3(10)	28.4(12)	18.5(10)	0.2(9)	6.2(8)	1.8(9)
C59	24.0(10)	22.4(11)	24.5(11)	-1.3(9)	7.4(9)	0.7(8)
C29	25.9(11)	24.6(11)	24.8(11)	5.0(9)	10.3(9)	2.7(9)
C41	22.2(10)	20.8(10)	22.3(10)	3.9(8)	4.8(8)	0.8(8)
C50	20.3(10)	21.3(10)	22.0(10)	4.6(8)	5.3(8)	2.4(8)
C31	21.3(10)	28.2(11)	22.5(10)	7.2(9)	6.8(8)	2.5(9)
C52	18.1(9)	23.7(11)	19.7(10)	-0.6(8)	4.1(8)	-1.1(8)
C7	26.6(11)	25.3(11)	22.7(10)	5.1(9)	8.9(9)	1.9(9)
C23	25.7(11)	26.1(12)	24.8(11)	0.9(9)	5.6(9)	-0.2(9)
C36	26.4(11)	29.8(12)	22.2(10)	5.2(9)	8.2(9)	1.8(9)
C8	27.5(11)	23.2(11)	21.2(10)	4.6(8)	6.8(9)	-2.4(9)
C2	30.2(12)	24.8(11)	24.4(11)	5.8(9)	11.4(9)	1.4(9)
C47	24.8(11)	25.9(11)	23.7(11)	-2.5(9)	7.1(9)	-4.4(9)
C42	20.0(10)	26.7(11)	16.4(9)	3.1(8)	2.8(8)	0.8(8)
C60	28.1(11)	26.5(12)	19.7(10)	-0.2(9)	5.3(9)	1.2(9)
C48	23.8(11)	33.0(13)	18.0(10)	-2.5(9)	5.7(8)	-2.4(9)
C53	22.2(10)	21.1(10)	22.0(10)	2.7(8)	5.8(8)	-2.2(8)
C1	29.9(12)	25.9(11)	23.4(11)	5.8(9)	9.7(9)	0.5(9)
C27	32.6(12)	26.9(12)	20.3(10)	2.6(9)	8.4(9)	-0.9(9)
C57	26.4(11)	28.5(12)	20.9(10)	-2.2(9)	8.3(9)	-2.6(9)
C21	34.7(12)	25.3(12)	26.2(11)	1.4(9)	11.8(10)	3.2(9)
C13	24.8(11)	37.0(13)	27.9(12)	10.8(10)	7.5(9)	1.0(10)
C3	36.3(13)	26.4(12)	27.7(12)	3.2(9)	10.7(10)	-3.4(10)
C12	29.2(12)	31.1(12)	24.2(11)	3.9(9)	5.4(9)	-3.9(10)
C33	25.4(11)	39.1(14)	29.1(12)	9.6(10)	10.6(10)	8.1(10)
C35	24.7(11)	39.0(14)	25.9(11)	4.8(10)	2.8(9)	-1.6(10)
C14	30.5(12)	41.5(14)	33.6(13)	12.9(11)	14.3(10)	6.6(11)
C6	31.9(12)	30.8(13)	30.7(12)	9.5(10)	15.0(10)	6.3(10)
C9	28.4(11)	27.7(12)	29.4(12)	7.6(9)	11.2(10)	2.8(9)
C18	27.9(12)	43.8(15)	26.7(12)	9.0(11)	7.0(10)	-0.1(11)
C28	34.1(12)	25.6(11)	24.6(11)	4.0(9)	13.8(10)	3.4(9)
C10	41.6(14)	24.9(12)	31.8(12)	1.6(10)	17.8(11)	2.5(10)
C22	33.3(12)	26.5(12)	23.9(11)	-2.9(9)	5.7(9)	-0.7(9)
C11	43.0(14)	28.2(12)	28.1(12)	-1.3(10)	12.8(11)	-4.8(10)
C4	52.1(16)	30.4(13)	24.2(12)	4.4(10)	14.2(11)	-0.5(11)
C34	22.4(11)	46.6(15)	30.8(12)	8.2(11)	5.9(10)	4.1(10)
C5	50.9(15)	28.4(13)	31.8(13)	5.6(10)	21.1(12)	5.4(11)
C17	31.0(13)	64.1(19)	30.1(13)	9.5(13)	3.2(11)	-6.8(13)
C15	28.5(13)	60.0(18)	37.7(14)	19.8(13)	12.6(11)	10.4(12)
C16	25.9(13)	74(2)	38.5(15)	21.4(15)	7.4(11)	5.3(13)
C61	36.3(15)	85(2)	36.9(15)	10.4(16)	10.8(12)	16.3(15)

Table S4. Bond Lengths for $(TOTA)_2 \cdot (F_4TCNQ)_2 \cdot CH_2Cl_2$.

Atom	Atom	Length/Å	Atom	Atom	Length/Å
Cl2	C61	1.764(3)	C38	C39	1.359(3)
Cl1	C61	1.763(4)	C51	C50	1.356(3)
F3	C39	1.346(2)	C51	C52	1.420(3)
F8	C50	1.346(2)	C56	C55	1.427(3)
F2	C41	1.345(2)	C39	C40	1.408(3)
F6	C53	1.349(2)	C40	C41	1.419(3)
F4	C38	1.348(2)	C55	C52	1.406(3)
F1	C42	1.346(2)	C55	C57	1.425(3)
F7	C51	1.346(2)	C49	C58	1.407(3)
F5	C54	1.346(2)	C49	C54	1.419(3)
O5	C24	1.369(3)	C49	C50	1.421(3)
O5	C30	1.376(3)	C46	C47	1.425(3)
O6	C26	1.381(3)	C46	C48	1.432(3)
O6	C36	1.370(3)	C58	C59	1.422(3)
O4	C20	1.383(3)	C58	C60	1.424(3)
O4	C32	1.372(3)	C32	C31	1.403(3)
O2	C8	1.372(3)	C32	C33	1.378(3)
O2	C2	1.376(3)	C24	C23	1.378(3)
O3	C12	1.387(3)	C30	C29	1.373(3)
O3	C18	1.370(3)	C54	C53	1.355(3)
O1	C14	1.368(3)	C29	C28	1.391(3)
O1	C6	1.381(3)	C41	C42	1.360(3)
N2	C19	1.371(3)	C31	C36	1.391(3)
N2	C25	1.377(3)	C52	C53	1.416(3)
N2	C31	1.377(3)	C7	C8	1.396(3)
N3	C45	1.155(3)	C7	C12	1.399(3)
N1	C7	1.375(3)	C23	C22	1.381(3)
N1	C1	1.375(3)	C36	C35	1.382(3)
N1	C13	1.381(3)	C8	C9	1.374(3)
N4	C44	1.151(3)	C2	C1	1.392(3)
N10	C59	1.152(3)	C2	C3	1.375(3)
N8	C56	1.150(3)	C1	C6	1.397(3)
N6	C47	1.150(3)	C27	C28	1.393(3)
N7	C57	1.151(3)	C21	C22	1.395(3)
N5	C48	1.153(3)	C13	C14	1.396(4)
N9	C60	1.153(3)	C13	C18	1.393(4)
C37	C38	1.415(3)	C3	C4	1.391(4)
C37	C46	1.414(3)	C12	C11	1.372(4)
C37	C42	1.417(3)	C33	C34	1.390(4)
C26	C25	1.398(3)	C35	C34	1.384(4)
C26	C27	1.371(3)	C14	C15	1.380(3)
C19	C20	1.397(3)	C6	C5	1.369(4)
C19	C24	1.400(3)	C9	C10	1.387(3)
C43	C44	1.425(3)	C18	C17	1.386(3)
C43	C40	1.419(3)	C10	C11	1.396(4)
C43	C45	1.421(3)	C4	C5	1.392(4)
C20	C21	1.369(3)	C17	C16	1.396(4)

Table S4. Bond Lengths for $(TOTA)_2{\cdot}(F_4TCNQ)_2{\cdot}CH_2Cl_2$**.**

**Atom**	**Atom**	**Length/Å**	**Atom**	**Atom**	**Length/Å**
C25	C30	1.395(3)	C15	C16	1.377(4)

C58	C49	C50	123.6(2)	C36	C35	C34	118.2(2)
C54	C49	C50	112.60(19)	O1	C14	C13	121.3(2)
C37	C46	C47	124.1(2)	O1	C14	C15	119.2(2)
C37	C46	C48	122.64(19)	C15	C14	C13	119.5(3)
C47	C46	C48	112.62(19)	O1	C6	C1	120.6(2)
C49	C58	C59	122.7(2)	C5	C6	O1	119.2(2)
C49	C58	C60	123.3(2)	C5	C6	C1	120.2(2)
C59	C58	C60	114.04(19)	C8	C9	C10	118.5(2)
O4	C32	C31	121.3(2)	O3	C18	C13	121.5(2)
O4	C32	C33	119.3(2)	O3	C18	C17	119.1(2)
C33	C32	C31	119.3(2)	C17	C18	C13	119.4(3)
O5	C24	C19	121.03(19)	C29	C28	C27	122.0(2)
O5	C24	C23	119.28(19)	C9	C10	C11	122.6(2)
C23	C24	C19	119.7(2)	C23	C22	C21	122.8(2)
O5	C30	C25	120.78(19)	C12	C11	C10	118.1(2)
C29	C30	O5	118.87(19)	C3	C4	C5	121.9(2)
C29	C30	C25	120.3(2)	C35	C34	C33	123.1(2)
F5	C54	C49	118.75(18)	C6	C5	C4	118.7(2)
F5	C54	C53	117.82(19)	C18	C17	C16	118.3(3)
C53	C54	C49	123.36(19)	C16	C15	C14	118.8(3)
N3	C45	C43	176.5(2)	C15	C16	C17	122.7(3)
N10	C59	C58	177.0(2)	Cl1	C61	Cl2	112.15(16)
C30	C29	C28	118.6(2)				

Table S5. Bond Angles for $(TOTA)_2 \cdot (F_4TCNQ)_2 \cdot CH_2Cl_2$.

Atom	Atom	Atom	Angle/°	Atom	Atom	Atom	Angle/°
C24	O5	C30	118.87(17)	F2	C41	C40	118.52(19)
C36	O6	C26	118.82(17)	F2	C41	C42	117.85(18)
C32	O4	C20	118.85(17)	C42	C41	C40	123.6(2)
C8	O2	C2	118.80(17)	F8	C50	C51	117.46(18)
C18	O3	C12	118.70(19)	F8	C50	C49	118.63(19)
C14	O1	C6	118.77(19)	C51	C50	C49	123.9(2)
C19	N2	C25	120.03(18)	N2	C31	C32	118.9(2)
C19	N2	C31	120.43(19)	N2	C31	C36	120.0(2)
C31	N2	C25	119.54(19)	C36	C31	C32	121.1(2)
C7	N1	C13	120.1(2)	C55	C52	C51	124.14(19)
C1	N1	C7	119.97(19)	C55	C52	C53	123.2(2)
C1	N1	C13	119.9(2)	C53	C52	C51	112.63(19)
C38	C37	C42	112.76(19)	N1	C7	C8	119.7(2)
C46	C37	C38	122.8(2)	N1	C7	C12	119.9(2)
C46	C37	C42	124.44(19)	C8	C7	C12	120.4(2)
O6	C26	C25	120.47(19)	C24	C23	C22	118.6(2)
C27	C26	O6	119.5(2)	O6	C36	C31	121.1(2)
C27	C26	C25	120.0(2)	O6	C36	C35	119.0(2)
N2	C19	C20	120.1(2)	C35	C36	C31	119.8(2)
N2	C19	C24	119.5(2)	O2	C8	C7	120.8(2)
C20	C19	C24	120.4(2)	O2	C8	C9	119.2(2)
C40	C43	C44	122.0(2)	C9	C8	C7	120.0(2)
C40	C43	C45	123.66(19)	O2	C2	C1	121.0(2)
C45	C43	C44	114.05(19)	C3	C2	O2	119.2(2)
O4	C20	C19	120.3(2)	C3	C2	C1	119.8(2)
C21	C20	O4	119.3(2)	N6	C47	C46	174.8(2)
C21	C20	C19	120.4(2)	F1	C42	C37	118.75(19)
N2	C25	C26	120.03(19)	F1	C42	C41	117.92(19)
N2	C25	C30	119.7(2)	C41	C42	C37	123.30(19)
C30	C25	C26	120.2(2)	N9	C60	C58	176.8(2)
F4	C38	C37	118.32(19)	N5	C48	C46	175.8(2)
F4	C38	C39	117.75(18)	F6	C53	C54	117.66(18)
C39	C38	C37	123.9(2)	F6	C53	C52	118.20(19)
F7	C51	C50	117.73(19)	C54	C53	C52	124.1(2)
F7	C51	C52	118.91(19)	N1	C1	C2	119.6(2)
C50	C51	C52	123.36(19)	N1	C1	C6	119.9(2)
N8	C56	C55	176.2(3)	C2	C1	C6	120.5(2)
F3	C39	C38	117.43(19)	C26	C27	C28	118.9(2)
F3	C39	C40	119.10(19)	N7	C57	C55	176.4(2)
C38	C39	C40	123.41(19)	C20	C21	C22	118.1(2)
N4	C44	C43	177.5(2)	N1	C13	C14	119.4(2)
C43	C40	C41	123.6(2)	N1	C13	C18	119.4(2)
C39	C40	C43	123.38(19)	C18	C13	C14	121.2(2)
C39	C40	C41	113.00(19)	C2	C3	C4	118.9(2)
C52	C55	C56	122.9(2)	O3	C12	C7	120.4(2)
C52	C55	C57	122.93(19)	C11	C12	O3	119.3(2)
C57	C55	C56	113.67(19)	C11	C12	C7	120.2(2)

Table S5. Bond Angles for $(TOTA)_2 \cdot (F_4TCNQ)_2 \cdot CH_2Cl_2$.

Atom	Atom	Atom	Angle/°	Atom	Atom	Atom	Angle/°
C58	C49	C54	123.80(19)	C32	C33	C34	118.4(2)

Table S6. Crystal data and structure refinement for $(TOTA)_4 \cdot (F_4BQ)$

Empirical formula	$C_{21}H_9F_2NO_4$
Formula weight	377.29
Temperature/K	100
Crystal system	triclinic
Space group	$P\bar{1}$
a/Å	9.0452(6)
b/Å	10.1512(7)
c/Å	10.1574(7)
α/°	112.442(5)
β/°	94.726(5)
γ/°	115.784(5)
Volume/Å^3	740.80(10)
Z	2
ρ_{calc} g/cm^3	1.691
μ/mm^{-1}	0.135
F(000)	384.0
Crystal size/mm^3	0.25 × 0.217 × 0.15
Radiation	MoK$_\alpha$ (λ = 0.71073)
2Θ range for data collection/°	4.972 to 55.152°
Index ranges	$-11 \leq h \leq 11$, $-13 \leq k \leq 13$, $-13 \leq l \leq 13$
Reflections collected	6260
Independent reflections	3312 [R_{int} = 0.0201, R_{sigma} = 0.0196]
Data/restraints/parameters	3312/0/253
Goodness-of-fit on F^2	1.034
Final R indexes [I ≥ 2σ(I)]	R_1 = 0.0382, wR_2 = 0.1005
Final R indexes [all data]	R_1 = 0.0500, wR_2 = 0.1094
Largest diff. peak/hole / e Å^{-3}	0.24/-0.27

Table S7. Fractional Atomic Coordinates (×10^4) and Equivalent Isotropic Displacement Parameters ($Å^2$×10^3) for $(TOTA)_4$·(F_4BQ). U_{eq} is defined as 1/3 of the trace of the orthogonalised U_{ij} tensor.

**Atom**	***x***	***y***	***z***	**U(eq)**
F2	7353.6(9)	8286.7(9)	1760.6(9)	22.96(19)
F1	4072.4(9)	7332.9(9)	436.4(9)	22.81(19)
O4	8219.1(11)	5933.8(12)	1352.3(11)	22.6(2)
O1	8378.2(11)	5885.6(11)	4647.9(11)	21.7(2)
O3	4311.8(11)	7510.3(11)	3583.1(10)	21.5(2)
O2	2662.0(11)	1740.9(11)	1254.8(11)	21.4(2)
N1	5209.6(13)	5071.7(13)	2943.6(12)	18.3(2)
C19	6760.7(16)	5510.5(15)	730.4(14)	18.7(3)
C7	3493.4(16)	4625.5(16)	2459.8(14)	18.6(3)
C21	4565.6(16)	6229.3(15)	248.3(14)	18.6(3)
C18	7908.5(16)	7075.2(16)	4912.1(14)	19.5(3)
C6	4265.7(16)	2179.1(16)	2061.5(14)	19.7(3)
C20	6177.1(16)	6695.6(15)	894.2(14)	18.8(3)
C8	2236.2(16)	2968.8(16)	1607.8(14)	19.6(3)
C13	6333.3(16)	6680.8(15)	4099.0(14)	18.6(3)
C12	3057.5(16)	5849.5(16)	2772.7(14)	19.4(3)
C14	5892.1(16)	7894.2(16)	4388.6(14)	19.5(3)
C1	5521.5(16)	3827.4(16)	2935.5(14)	18.8(3)
C11	1382.0(17)	5429.5(17)	2226.7(15)	22.6(3)
C9	547.5(16)	2521.3(17)	1056.2(15)	22.9(3)
C3	7464.9(17)	3022.0(17)	3653.8(15)	22.9(3)
C2	7107.5(16)	4245.5(16)	3748.1(14)	19.3(3)
C5	4600.7(17)	942.0(17)	1936.1(15)	22.8(3)
C10	142.3(16)	3763.2(17)	1362.4(15)	23.7(3)
C4	6213.2(17)	1377.2(17)	2730.7(16)	24.6(3)
C15	7032.4(17)	9511.2(16)	5441.5(15)	22.1(3)
C16	8619.3(17)	9891.7(16)	6235.4(15)	23.2(3)
C17	9056.3(16)	8679.5(17)	5985.3(15)	22.3(3)

Table S8. Anisotropic Displacement Parameters (Å^2×10^3) for $(TOTA)_4·(F_4BQ)$. The Anisotropic displacement factor exponent takes the form: $-2\pi^2[h^2a^{*2}U_{11}+2hka^*b^*U_{12}+…]$.

**Atom**	**U_{11}**	**U_{22}**	**U_{33}**	**U_{23}**	**U_{13}**	**U_{12}**
F2	17.9(4)	13.9(4)	25.8(4)	3.5(3)	-0.6(3)	5.4(3)
F1	21.5(4)	19.4(4)	29.3(4)	9.1(3)	4.1(3)	14.2(3)
O4	15.8(4)	23.6(5)	24.9(5)	8.8(4)	1.3(3)	10.0(4)
O1	15.0(4)	19.0(5)	26.2(5)	7.0(4)	0.1(3)	8.7(4)
O3	17.7(5)	17.3(5)	25.0(5)	5.9(4)	0.5(4)	9.8(4)
O2	17.1(4)	16.7(4)	25.4(5)	6.5(4)	0.3(4)	8.4(4)
N1	15.0(5)	16.1(5)	20.6(5)	6.0(4)	1.0(4)	8.2(4)
C19	15.4(6)	19.3(6)	19.3(6)	7.6(5)	2.4(5)	8.7(5)
C7	15.1(6)	19.5(6)	20.0(6)	8.2(5)	3.1(5)	8.9(5)
C21	19.3(6)	17.3(6)	21.2(6)	8.0(5)	4.8(5)	12.0(5)
C18	17.2(6)	19.9(6)	20.7(6)	8.6(5)	4.6(5)	9.8(5)
C6	16.3(6)	20.1(6)	21.2(6)	8.8(5)	3.8(5)	9.2(5)
C20	16.3(6)	14.4(6)	19.6(6)	4.6(5)	2.4(5)	6.3(5)
C8	18.1(6)	19.3(6)	21.8(6)	9.2(5)	4.7(5)	10.4(5)
C13	15.7(6)	17.0(6)	19.2(6)	6.7(5)	3.2(5)	7.0(5)
C12	17.0(6)	17.7(6)	20.2(6)	6.7(5)	3.2(5)	8.5(5)
C14	15.6(6)	20.4(6)	21.2(6)	8.6(5)	3.6(5)	9.3(5)
C1	18.0(6)	19.1(6)	21.5(6)	9.4(5)	5.5(5)	11.3(5)
C11	18.5(6)	24.2(7)	27.2(7)	10.7(6)	5.1(5)	13.8(5)
C9	16.5(6)	21.6(6)	25.5(7)	9.4(5)	2.9(5)	7.7(5)
C3	20.2(6)	25.1(7)	25.7(7)	11.2(6)	4.2(5)	14.4(5)
C2	16.9(6)	19.1(6)	19.9(6)	7.3(5)	4.0(5)	9.3(5)
C5	22.3(7)	18.1(6)	26.1(7)	8.3(5)	5.0(5)	10.7(5)
C10	15.5(6)	26.1(7)	27.4(7)	10.6(6)	3.5(5)	10.8(5)
C4	24.6(7)	23.1(7)	30.4(7)	11.7(6)	7.1(5)	16.1(6)
C15	21.2(6)	18.5(6)	24.3(7)	7.6(5)	5.4(5)	10.3(5)
C16	19.8(6)	18.3(6)	22.8(6)	6.0(5)	3.1(5)	6.5(5)
C17	16.0(6)	22.2(6)	22.7(6)	8.3(5)	1.5(5)	7.5(5)

Table S9. Bond Lengths for $(TOTA)_4{\cdot}(F_4BQ)$.

Atom	Atom	Length/Å
F2	C20	1.3345(14)
F1	C21	1.3326(14)
O4	C19	1.2191(15)
O1	C18	1.3887(15)
O1	C2	1.3888(15)
O3	C12	1.3892(15)
O3	C14	1.3941(15)
O2	C6	1.3921(15)
O2	C8	1.3892(15)
N1	C7	1.3961(16)
N1	C13	1.4050(16)
N1	C1	1.4062(16)
C19	C21[1]	1.4718(18)
C19	C20	1.4724(17)
C7	C8	1.3903(18)
C7	C12	1.3948(17)
C21	C20	1.3358(18)
C18	C13	1.3892(17)
C18	C17	1.3804(19)
C6	C1	1.3872(18)
C6	C5	1.3785(18)
C8	C9	1.3837(17)
C13	C14	1.3905(17)
C12	C11	1.3803(18)
C14	C15	1.3809(18)
C1	C2	1.3883(17)
C11	C10	1.3940(18)
C9	C10	1.3935(18)
C3	C2	1.3855(18)
C3	C4	1.3907(19)
C5	C4	1.3954(18)
C15	C16	1.3961(18)
C16	C17	1.3913(18)

[1]1-X,1-Y,-Z

Table S10. Bond Angles for (TOTA)$_4$·(F$_4$BQ).

Atom	Atom	Atom	Angle/°	Atom	Atom	Atom	Angle/°
C18	O1	C2	117.35(10)	C9	C8	C7	120.37(12)
C12	O3	C14	117.33(10)	C18	C13	N1	120.16(11)
C8	O2	C6	117.40(10)	C18	C13	C14	120.05(12)
C7	N1	C13	117.00(10)	C14	C13	N1	119.74(11)
C7	N1	C1	116.86(10)	O3	C12	C7	120.63(11)
C13	N1	C1	116.57(10)	C11	C12	O3	118.92(11)
O4	C19	C21^{1}	122.81(12)	C11	C12	C7	120.39(12)
O4	C19	C20	122.86(12)	C13	C14	O3	120.51(11)
C21^{1}	C19	C20	114.33(11)	C15	C14	O3	118.76(11)
C8	C7	N1	120.01(11)	C15	C14	C13	120.69(12)
C8	C7	C12	120.14(11)	C6	C1	N1	119.75(11)
C12	C7	N1	119.77(11)	C6	C1	C2	120.08(12)
F1	C21	C19^{1}	115.84(11)	C2	C1	N1	120.10(11)
F1	C21	C20	121.38(11)	C12	C11	C10	118.65(12)
C20	C21	C19^{1}	122.76(11)	C8	C9	C10	118.63(12)
O1	C18	C13	121.19(11)	C2	C3	C4	118.89(12)
C17	C18	O1	118.67(11)	C1	C2	O1	121.24(11)
C17	C18	C13	120.13(12)	C3	C2	O1	118.50(11)
C1	C6	O2	120.73(11)	C3	C2	C1	120.25(12)
C5	C6	O2	118.63(11)	C6	C5	C4	118.79(12)
C5	C6	C1	120.60(12)	C9	C10	C11	121.80(12)
F2	C20	C19	115.92(11)	C3	C4	C5	121.31(12)
F2	C20	C21	121.19(11)	C14	C15	C16	118.53(12)
C21	C20	C19	122.88(11)	C17	C16	C15	121.30(12)
O2	C8	C7	120.69(11)	C18	C17	C16	119.26(12)
C9	C8	O2	118.91(11)				

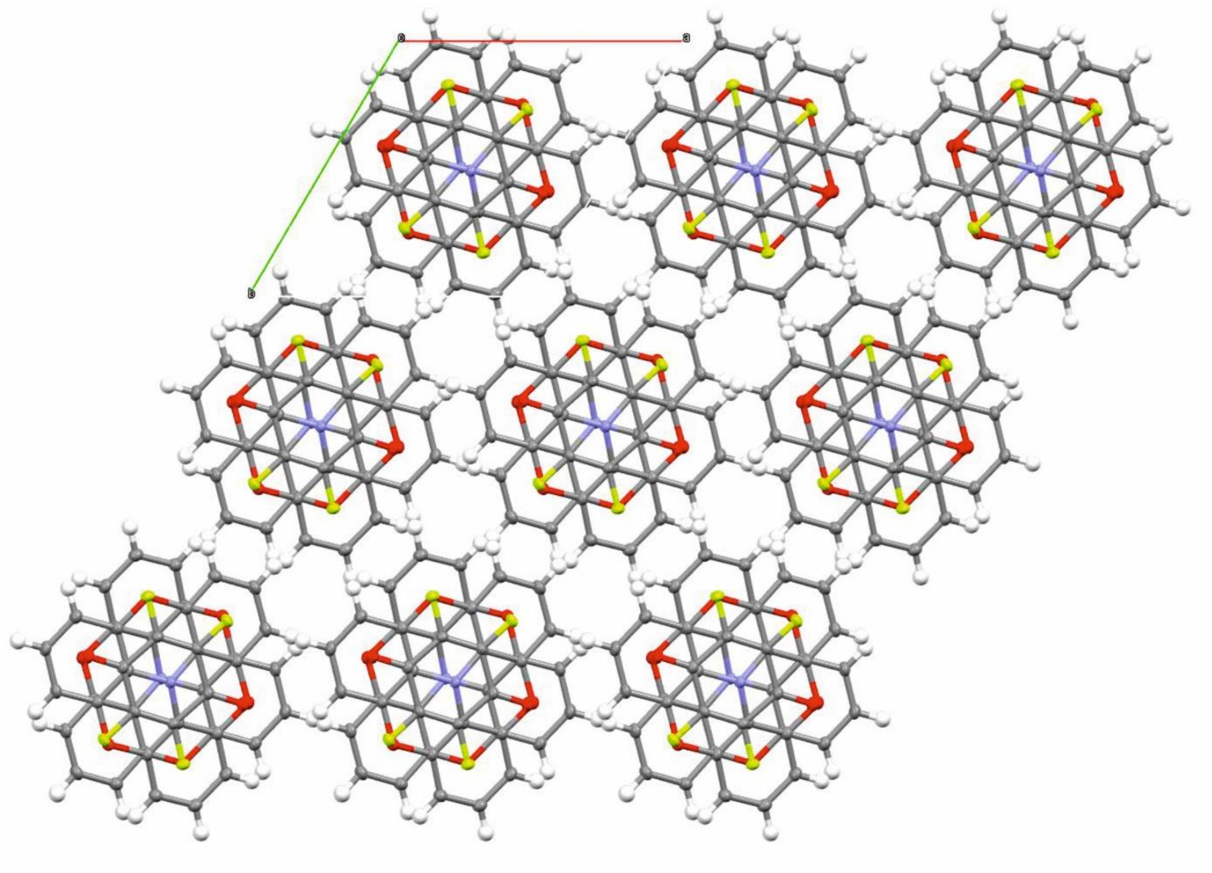

Figure S5. Packing of $(TOTA)_2(F_4BQ)$ along the ab plane, viewing down the *c* axis.

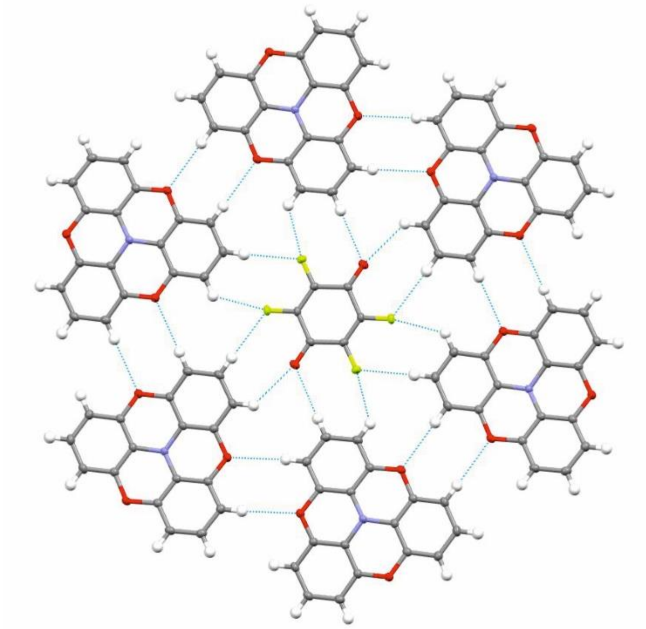

Figure S6. Surrounding of the F_4BQ acceptor in crystalline TOTA·F_4BQ with intermolecular H-bonding interactions indicated by blue broken lines.

Table S11. Crystal data and structure refinement for $(PAA)_4{\cdot}(F_4TCNQ)$

Empirical formula	$C_{70}H_{56}F_2N_6Si_4$
Formula weight	1131.56
Temperature/K	100
Crystal system	monoclinic
Space group	*C*2/*c*
a/Å	26.921(4)
b/Å	19.255(2)
c/Å	25.544(3)
α/°	90
β/°	101.364(12)
γ/°	90
Volume/Å^3	12982(3)
Z	8
ρ_{calc} g/cm^3	1.158
μ/mm^{-1}	1.248
F(000)	4736.0
Crystal size/mm^3	0.1 × 0.06 × 0.04
Radiation	Cu K_α (λ = 1.54186)
2Θ range for data collection/°	6.698 to 135.288°
Index ranges	$-32 \le h \le 30$, $-21 \le k \le 22$, $-26 \le l \le 30$
Reflections collected	45229
Independent reflections	11390 [R_{int} = 0.1041, R_{sigma} = 0.0952]
Data/restraints/parameters	11390/30/751
Goodness-of-fit on F^2	1.033
Final R indexes [I ≥ 2σ (I)]	R_1 = 0.0835, wR_2 = 0.1720
Final R indexes [all data]	R_1 = 0.1707, wR_2 = 0.2267
Largest diff. peak/hole / e Å^{-3}	0.38/-0.47

Table S12. Fractional Atomic Coordinates (×10^4) and Equivalent Isotropic Displacement Parameters (Å^2×10^3) for $(PAA)_4$·(F_4TCNQ). U_{eq} is defined as 1/3 of the trace of the orthogonalised U_{IJ} tensor.

Atom	*x*	*y*	*z*	U(eq)
Si2	2386.0(6)	5131.9(9)	6537.5(7)	36.0(4)
Si4	5834.3(7)	2201.3(10)	3305.4(7)	39.8(4)
Si1	5490.4(7)	792.5(10)	6746.9(7)	44.0(5)
Si3	2783.0(7)	6586.5(10)	3270.7(7)	40.2(5)
F2	1937.9(11)	3652.9(16)	5090.6(12)	31.2(7)
F1	2404.3(11)	3026.2(16)	5963.4(12)	30.9(7)
N4	4202.7(14)	2460(2)	5789.3(16)	20.4(10)
N6	4097.0(14)	4908(2)	4214.3(16)	20.5(9)
N3	3525.0(15)	3588(2)	5745.5(16)	21.9(10)
N5	4776.9(15)	3781(2)	4222.4(16)	20.2(9)
C19	4052.3(18)	2408(3)	4832(2)	22.7(12)
C32	4373(2)	1837(3)	4838(2)	26.5(13)
C17	3632.5(17)	3305(3)	5304.9(19)	18.3(11)
C9	3656.2(18)	3607(3)	6700.0(19)	19.9(11)
C70	2211.4(19)	3078(3)	5040(2)	25.4(12)
C41	3914.1(18)	4889(3)	3252.8(19)	21.4(11)
C10	3763.7(18)	3320(3)	6220(2)	20.6(11)
C51	4952.5(18)	3766(3)	5185(2)	19.6(11)
C29	3879(2)	2373(3)	3851(2)	25.9(12)
N2	3584(2)	743(3)	6004(2)	49.3(15)
C7	4256.3(18)	2789(3)	7208(2)	23.0(12)
C60	5029(2)	4074(3)	6654(2)	25.9(12)
C39	4359.0(18)	4066(3)	2787(2)	22.1(12)
C38	4616.0(18)	3792(3)	3264.4(19)	19.8(11)
C6	4362.0(18)	2499(3)	6749(2)	20.7(11)
C57	4098(2)	5468(3)	6137(2)	25.2(12)
C58	4435.8(19)	4908(3)	6153(2)	24.8(12)
C64	5279.7(19)	3209(3)	5205(2)	24.3(12)
C26	3226.5(19)	3540(3)	3809(2)	25.1(12)
C54	4263.5(18)	4930(3)	5175.5(19)	19.8(11)
C5	4720.4(19)	1947(3)	6765(2)	23.2(12)
C40	4005.4(18)	4610(3)	2784(2)	27.2(13)
C42	4183.5(18)	4625(3)	3754(2)	20.5(11)
C62	5446.5(19)	3213(3)	6158(2)	27.5(13)
C55	3932.5(19)	5483(3)	5181(2)	23.2(12)
C22	3388.8(18)	3591(3)	4792(2)	20.3(11)
C61	5113.9(19)	3784(3)	6162(2)	24.1(12)
C13	2946(2)	4571(3)	6621(2)	30.0(13)
C18	3968.2(17)	2721(3)	5326(2)	19.7(11)
C52	4858.9(18)	4062(3)	5663(2)	21.2(11)
C12	3279.7(18)	4138(3)	6668(2)	22.7(12)
C49	4349.6(17)	4632(3)	4666.0(19)	19.4(11)
C23	3074.4(19)	4179(3)	4753(2)	25.3(12)
C36	5294(2)	2794(3)	3268(2)	31.0(13)
C50	4689.7(17)	4060(3)	4674.7(19)	18.0(11)
C8	3902.3(18)	3339(3)	7183(2)	24.3(12)

Table S12. Fractional Atomic Coordinates (×10^4) and Equivalent Isotropic Displacement Parameters (Å^2×10^3) for $(PAA)_4 \cdot (F_4TCNQ)$. U_{eq} is defined as 1/3 of the trace of the orthogonalised U_{IJ} tensor.

**Atom**	***x***	***y***	***z***	**U(eq)**
C53	4520.3(18)	4634(3)	5660.4(19)	19.9(11)
C67	2979.6(19)	2048(3)	6483(2)	29.1(13)
C59	4699.6(19)	4608(3)	6648(2)	24.6(12)
C20	3799.9(18)	2682(3)	4337.3(19)	21.1(11)
C68	2764.9(18)	2156(3)	5489(2)	23.4(12)
C69	2450.2(19)	2769(3)	5490(2)	23.1(12)
C11	4108.9(17)	2762(3)	6241.9(18)	18.4(11)
C21	3467.4(18)	3271(3)	4312(2)	23.8(12)
C56	3854(2)	5749(3)	5663(2)	28.0(13)
C25	2915.1(19)	4128(3)	3793(2)	27.9(13)
C28	3623(2)	2659(3)	3355(2)	32.9(14)
C24	2845.7(19)	4445(3)	4264(2)	28.2(13)
C63	5525(2)	2937(3)	5685(2)	29.4(13)
C31	4456(2)	1542(3)	4369(2)	34.6(14)
C4	5032(2)	1489(3)	6781(2)	29.2(13)
C66	3012(2)	1833(3)	5950(2)	27.7(13)
C27	3311(2)	3212(3)	3330(2)	29.6(13)
C37	4985(2)	3244(3)	3270(2)	25.1(12)
C45	3255(2)	5902(3)	3249(2)	30.5(13)
C43	4530.4(17)	4065(3)	3762.4(19)	17.8(11)
C44	3559.5(19)	5443(3)	3251(2)	24.2(12)
C30	4213(2)	1807(3)	3884(2)	32.7(14)
C48	2482(3)	6392(4)	3853(3)	69(2)
C65	3327(2)	1225(3)	5952(2)	37.2(15)
C33	6398(2)	2755(3)	3296(3)	46.7(17)
C35	5719(3)	1606(4)	2719(3)	54.8(19)
C46	3113(3)	7428(4)	3351(3)	60(2)
C16	2418(3)	5624(4)	7167(3)	59(2)
C34	5892(3)	1691(5)	3931(3)	83(3)
C15	1821(2)	4560(4)	6393(3)	57(2)
C47	2312(2)	6570(4)	2631(3)	55(2)
C3	5977(3)	781(4)	7369(3)	64(2)
C2	5136(4)	-38(4)	6665(4)	85(3)
C14	2398(3)	5728(4)	5966(3)	75(3)
C1	5772(4)	999(5)	6156(4)	109(4)
N1	2974.1(19)	2167(3)	6924(2)	40.7(13)

Table S13. Anisotropic Displacement Parameters ($Å^2×10^3$) for $(PAA)_4·(F_4TCNQ)$. The Anisotropic displacement factor exponent takes the form: $-2\pi^2[h^2a^{*2}U_{11}+2hka^*b^*U_{12}+…]$.

**Atom**	U_{11}	U_{22}	U_{33}	U_{23}	U_{13}	U_{12}
Si2	33.1(9)	38.9(10)	36.4(10)	-1.7(8)	7.5(7)	13.5(8)
Si4	42.9(10)	41.1(11)	38.0(10)	4.9(8)	14.8(8)	18.8(8)
Si1	58.0(11)	39.5(11)	37.2(10)	5.5(8)	15.7(8)	23.9(9)
Si3	43.4(10)	48.3(11)	28.3(9)	5.6(8)	5.7(7)	24.0(9)
F2	34.9(17)	28.1(18)	31.4(18)	-3.5(14)	8.3(14)	0.7(14)
F1	36.7(18)	33.4(19)	23.9(17)	-3.8(14)	9.3(13)	-4.0(15)
N4	18(2)	28(3)	16(2)	-2.4(19)	2.8(17)	-5.5(18)
N6	17(2)	27(3)	17(2)	1.8(19)	1.9(16)	-1.4(18)
N3	23(2)	21(2)	22(2)	-4.3(19)	4.6(18)	-3.8(18)
N5	19(2)	20(2)	22(2)	0.0(19)	4.9(17)	-2.9(18)
C19	23(3)	27(3)	19(3)	-2(2)	6(2)	-7(2)
C32	34(3)	31(3)	14(3)	-9(2)	3(2)	1(3)
C17	18(2)	20(3)	17(3)	0(2)	1.6(19)	-7(2)
C9	17(2)	26(3)	17(3)	-5(2)	3.8(19)	-1(2)
C70	21(3)	22(3)	34(3)	-1(2)	7(2)	-3(2)
C41	23(3)	25(3)	17(3)	-3(2)	7(2)	-3(2)
C10	21(2)	21(3)	20(3)	-2(2)	3(2)	-4(2)
C51	18(2)	20(3)	21(3)	2(2)	3(2)	-4(2)
C29	31(3)	25(3)	23(3)	-4(2)	7(2)	-9(2)
N2	53(3)	46(4)	47(4)	4(3)	5(3)	8(3)
C7	22(3)	26(3)	22(3)	-3(2)	5(2)	-3(2)
C60	35(3)	29(3)	14(3)	7(2)	4(2)	-7(3)
C39	22(3)	30(3)	15(3)	-1(2)	6(2)	3(2)
C38	25(3)	17(3)	17(3)	-2(2)	4(2)	-3(2)
C6	21(2)	23(3)	18(3)	-4(2)	3(2)	-1(2)
C57	33(3)	24(3)	21(3)	-6(2)	10(2)	1(2)
C58	25(3)	30(3)	19(3)	0(2)	2(2)	-9(2)
C64	29(3)	27(3)	17(3)	1(2)	3(2)	-3(2)
C26	24(3)	33(3)	18(3)	2(2)	1(2)	-7(2)
C54	20(3)	24(3)	16(3)	-2(2)	4.5(19)	-2(2)
C5	27(3)	25(3)	17(3)	0(2)	3(2)	1(2)
C40	19(3)	36(3)	27(3)	4(3)	4(2)	-3(2)
C42	19(2)	20(3)	22(3)	1(2)	4(2)	1(2)
C62	27(3)	30(3)	24(3)	11(2)	1(2)	1(2)
C55	26(3)	20(3)	24(3)	3(2)	5(2)	2(2)
C22	20(2)	19(3)	22(3)	4(2)	3(2)	-2(2)
C61	26(3)	28(3)	18(3)	4(2)	4(2)	-6(2)
C13	33(3)	36(3)	20(3)	-7(3)	5(2)	-6(3)
C18	15(2)	22(3)	22(3)	1(2)	4.3(19)	-3(2)
C52	24(3)	18(3)	21(3)	-1(2)	4(2)	-5(2)
C12	20(3)	30(3)	19(3)	-3(2)	6(2)	3(2)
C49	15(2)	22(3)	20(3)	-4(2)	2(2)	-5(2)
C23	26(3)	25(3)	25(3)	2(2)	6(2)	-6(2)
C36	45(3)	32(3)	16(3)	-4(2)	5(2)	-2(3)
C50	18(2)	18(3)	17(3)	7(2)	2.6(19)	-3(2)
C8	18(3)	34(3)	21(3)	-4(2)	5(2)	-6(2)

Table S13. Anisotropic Displacement Parameters (Å^2×10^3) for $(PAA)_4\cdot(F_4TCNQ)$. The Anisotropic displacement factor exponent takes the form: $-2\pi^2[h^2a^{*2}U_{11}+2hka^*b^*U_{12}+\ldots]$.

Atom	U_{11}	U_{22}	U_{33}	U_{23}	U_{13}	U_{12}
C53	25(3)	22(3)	12(3)	0(2)	4(2)	-7(2)
C67	24(3)	33(3)	28(3)	0(3)	0(2)	-8(2)
C59	28(3)	27(3)	19(3)	-3(2)	6(2)	-7(2)
C20	23(3)	23(3)	17(3)	0(2)	5(2)	-6(2)
C68	21(3)	23(3)	27(3)	-3(2)	7(2)	-3(2)
C69	24(3)	25(3)	22(3)	-7(2)	7(2)	-5(2)
C11	19(2)	24(3)	13(3)	0(2)	4.3(19)	-3(2)
C21	22(3)	28(3)	20(3)	2(2)	2(2)	-9(2)
C56	32(3)	27(3)	26(3)	-6(2)	9(2)	3(2)
C25	24(3)	30(3)	26(3)	6(3)	-3(2)	-10(2)
C28	29(3)	50(4)	20(3)	-4(3)	6(2)	-12(3)
C24	23(3)	32(3)	29(3)	5(3)	4(2)	-5(2)
C63	29(3)	31(3)	26(3)	7(3)	1(2)	6(2)
C31	35(3)	42(4)	28(3)	-8(3)	9(2)	7(3)
C4	36(3)	31(3)	20(3)	3(3)	4(2)	-2(3)
C66	25(3)	28(3)	29(3)	1(2)	3(2)	-9(2)
C27	26(3)	43(4)	18(3)	-2(3)	2(2)	-9(3)
C37	33(3)	19(3)	24(3)	-5(2)	7(2)	0(2)
C45	38(3)	36(4)	18(3)	7(3)	6(2)	0(3)
C43	16(2)	17(3)	20(3)	0(2)	3.1(19)	0(2)
C44	27(3)	27(3)	19(3)	10(2)	5(2)	1(2)
C30	39(3)	39(4)	24(3)	-11(3)	15(2)	-8(3)
C48	71(5)	89(6)	50(5)	14(4)	24(4)	44(5)
C65	33(3)	39(4)	37(4)	6(3)	-1(3)	-3(3)
C33	37(3)	51(4)	48(4)	-13(3)	-1(3)	18(3)
C35	56(4)	46(4)	71(5)	-8(4)	36(4)	4(3)
C46	77(5)	61(5)	38(4)	3(4)	3(4)	31(4)
C16	55(4)	56(5)	69(5)	-25(4)	15(4)	10(4)
C34	84(6)	106(7)	68(6)	39(5)	35(5)	58(6)
C15	40(4)	59(5)	69(5)	-10(4)	1(3)	17(3)
C47	47(4)	73(5)	44(4)	4(4)	11(3)	24(4)
C3	51(4)	69(5)	72(4)	6(4)	16(3)	23(4)
C2	108(6)	47(5)	92(7)	-16(4)	-3(5)	17(4)
C14	65(5)	81(6)	86(6)	33(5)	28(5)	40(5)
C1	139(8)	117(8)	89(5)	60(6)	72(6)	97(6)
N1	45(3)	50(4)	27(3)	4(3)	8(2)	-10(3)

Table S14. Bond Lengths for $(PAA)_4{\cdot}(F_4TCNQ)$.

Atom	Atom	Length/Å	Atom	Atom	Length/Å
Si2	C13	1.833(6)	C29	C30	1.403(8)
Si2	C16	1.854(7)	N2	C65	1.149(8)
Si2	C15	1.854(7)	C7	C6	1.377(7)
Si2	C14	1.862(8)	C7	C8	1.418(7)
Si4	C36	1.836(6)	C60	C61	1.434(7)
Si4	C33	1.859(7)	C60	C59	1.356(7)
Si4	C35	1.863(7)	C39	C38	1.384(7)
Si4	C34	1.855(7)	C39	C40	1.414(7)
Si1	C4	1.835(6)	C38	C37	1.448(7)
Si1	C3	1.849(7)	C38	C43	1.436(7)
Si1	C2	1.852(9)	C6	C5	1.430(7)
Si1	C1	1.862(8)	C6	C11	1.431(7)
Si3	C45	1.839(6)	C57	C58	1.405(7)
Si3	C48	1.866(7)	C57	C56	1.368(7)
Si3	C46	1.841(8)	C58	C53	1.424(7)
Si3	C47	1.861(7)	C58	C59	1.443(7)
F2	C70	1.350(6)	C64	C63	1.376(7)
F1	C69	1.335(6)	C26	C21	1.418(7)
N4	C18	1.325(6)	C26	C25	1.405(8)
N4	C11	1.361(6)	C26	C27	1.435(7)
N6	C42	1.358(6)	C54	C55	1.390(7)
N6	C49	1.329(6)	C54	C49	1.482(7)
N3	C17	1.332(6)	C54	C53	1.414(7)
N3	C10	1.358(6)	C5	C4	1.214(7)
N5	C50	1.336(6)	C42	C43	1.424(7)
N5	C43	1.346(6)	C62	C61	1.418(7)
C19	C32	1.395(7)	C62	C63	1.374(8)
C19	C18	1.457(7)	C55	C56	1.388(7)
C19	C20	1.413(7)	C22	C23	1.405(7)
C32	C31	1.383(7)	C22	C21	1.424(7)
C17	C22	1.454(7)	C61	C52	1.428(7)
C17	C18	1.438(7)	C13	C12	1.214(7)
C9	C10	1.425(7)	C52	C53	1.428(7)
C9	C12	1.430(7)	C49	C50	1.430(7)
C9	C8	1.381(7)	C23	C24	1.379(7)
C70	C68[1]	1.438(7)	C36	C37	1.201(7)
C70	C69	1.340(7)	C67	C66	1.441(8)
C41	C40	1.377(7)	C67	N1	1.154(7)
C41	C42	1.434(7)	C20	C21	1.439(7)
C41	C44	1.431(7)	C68	C69	1.453(7)
C10	C11	1.415(7)	C68	C66	1.381(7)
C51	C64	1.383(7)	C25	C24	1.394(8)
C51	C52	1.416(7)	C28	C27	1.351(8)
C51	C50	1.468(7)	C31	C30	1.381(8)
C29	C20	1.431(7)	C66	C65	1.446(8)
C29	C28	1.429(7)	C45	C44	1.205(7)

[1]1/2-X,1/2-Y,1-Z

Table S15. Bond Angles for $(PAA)_4 \cdot (F_4TCNQ)$.

Atom	Atom	Atom	Angle/°
C13	Si2	C16	107.6(3)
C13	Si2	C15	107.3(3)
C13	Si2	C14	108.3(3)
C16	Si2	C15	111.8(3)
C16	Si2	C14	111.1(4)
C15	Si2	C14	110.5(4)
C36	Si4	C33	106.5(3)
C36	Si4	C35	109.6(3)
C36	Si4	C34	107.8(3)
C33	Si4	C35	110.5(3)
C34	Si4	C33	112.6(4)
C34	Si4	C35	109.8(4)
C4	Si1	C3	109.7(3)
C4	Si1	C2	107.4(3)
C4	Si1	C1	105.5(3)
C3	Si1	C2	110.4(4)
C3	Si1	C1	111.5(4)
C2	Si1	C1	112.1(5)
C45	Si3	C48	106.9(3)
C45	Si3	C46	108.2(3)
C45	Si3	C47	108.4(3)
C46	Si3	C48	111.4(4)
C46	Si3	C47	110.1(3)
C47	Si3	C48	111.5(3)
C18	N4	C11	117.5(4)
C49	N6	C42	116.5(4)
C17	N3	C10	117.2(4)
C50	N5	C43	116.9(4)
C32	C19	C18	121.1(5)
C32	C19	C20	119.5(5)
C20	C19	C18	119.4(5)
C31	C32	C19	121.3(5)
N3	C17	C22	118.2(5)
N3	C17	C18	121.8(4)
C18	C17	C22	119.9(5)
C10	C9	C12	119.3(4)
C8	C9	C10	118.7(5)
C8	C9	C12	121.9(5)
F2	C70	C68^1	118.2(5)
C69	C70	F2	117.5(5)
C69	C70	C68^1	124.4(5)
C40	C41	C42	119.4(5)
C40	C41	C44	121.4(5)
C44	C41	C42	119.1(5)
N3	C10	C9	118.7(5)
N3	C10	C11	121.0(5)
C11	C10	C9	120.3(5)
C55	C54	C53	120.3(5)
C53	C54	C49	118.6(5)
C4	C5	C6	178.7(6)
C41	C40	C39	121.4(5)
N6	C42	C41	119.2(4)
N6	C42	C43	121.0(4)
C43	C42	C41	119.8(5)
C63	C62	C61	120.9(5)
C56	C55	C54	120.0(5)
C23	C22	C17	121.8(5)
C23	C22	C21	118.6(5)
C21	C22	C17	119.7(5)
C62	C61	C60	121.3(5)
C62	C61	C52	118.5(5)
C52	C61	C60	120.2(5)
C12	C13	Si2	172.7(5)
N4	C18	C19	119.3(5)
N4	C18	C17	121.0(5)
C17	C18	C19	119.6(4)
C51	C52	C61	118.9(5)
C51	C52	C53	121.9(5)
C53	C52	C61	119.2(5)
C13	C12	C9	176.9(6)
N6	C49	C54	117.8(4)
N6	C49	C50	122.5(5)
C50	C49	C54	119.7(4)
C24	C23	C22	121.2(5)
C37	C36	Si4	171.7(5)
N5	C50	C51	118.5(4)
N5	C50	C49	121.1(4)
C49	C50	C51	120.4(5)
C9	C8	C7	121.2(5)
C58	C53	C52	119.6(5)
C54	C53	C58	119.3(5)
C54	C53	C52	121.1(5)
N1	C67	C66	174.1(7)
C60	C59	C58	121.4(5)
C19	C20	C29	119.5(5)
C19	C20	C21	121.4(5)
C29	C20	C21	119.1(5)
C70^1	C68	C69	112.9(5)
C66	C68	C70^1	124.0(5)
C66	C68	C69	123.2(5)
F1	C69	C70	119.8(5)
F1	C69	C68	117.5(5)
C70	C69	C68	122.7(5)
N4	C11	C10	121.4(4)

Table S15. Bond Angles for $(PAA)_4\cdot(F_4TCNQ)$.

Atom	Atom	Atom	Angle/°	Atom	Atom	Atom	Angle/°
C64	C51	C52	120.0(5)	N4	C11	C6	118.9(5)
C64	C51	C50	121.6(5)	C10	C11	C6	119.7(4)
C52	C51	C50	118.3(5)	C26	C21	C22	120.1(5)
C28	C29	C20	118.8(5)	C26	C21	C20	119.9(5)
C30	C29	C20	118.3(5)	C22	C21	C20	119.9(5)
C30	C29	C28	122.9(5)	C57	C56	C55	120.5(5)
C6	C7	C8	121.0(5)	C24	C25	C26	120.6(5)
C59	C60	C61	120.2(5)	C27	C28	C29	122.2(5)
C38	C39	C40	120.4(5)	C23	C24	C25	120.5(5)
C39	C38	C37	120.6(5)	C62	C63	C64	120.4(5)
C39	C38	C43	120.1(5)	C30	C31	C32	119.8(6)
C43	C38	C37	119.2(5)	C5	C4	Si1	175.4(5)
C7	C6	C5	121.8(5)	C67	C66	C65	112.0(5)
C7	C6	C11	119.0(5)	C68	C66	C67	124.6(5)
C5	C6	C11	119.1(5)	C68	C66	C65	123.4(5)
C56	C57	C58	121.7(5)	C28	C27	C26	120.5(5)
C57	C58	C53	118.2(5)	C36	C37	C38	179.0(6)
C57	C58	C59	122.5(5)	C44	C45	Si3	177.6(5)
C53	C58	C59	119.3(5)	N5	C43	C38	119.2(4)
C63	C64	C51	121.3(5)	N5	C43	C42	122.0(5)
C21	C26	C27	119.4(5)	C42	C43	C38	118.8(4)
C25	C26	C21	119.0(5)	C45	C44	C41	179.0(6)
C25	C26	C27	121.6(5)	C31	C30	C29	121.6(5)
C55	C54	C49	121.1(5)	N2	C65	C66	173.7(7)

[1]1/2-X,1/2-Y,1-Z

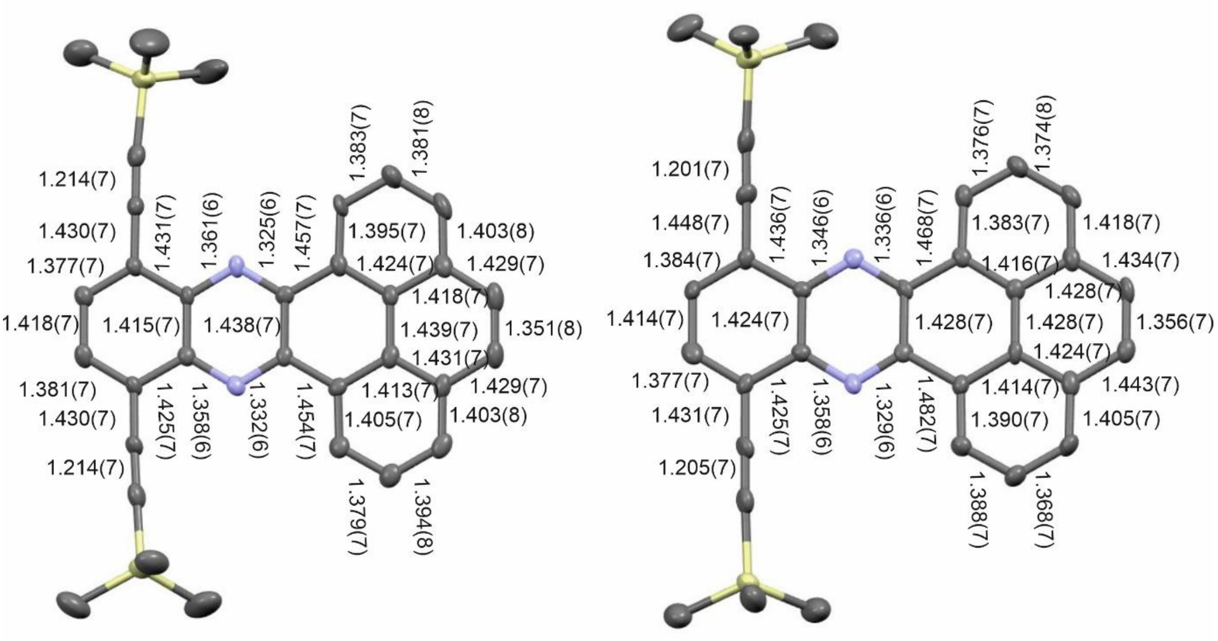

Figure S7. Comparison of the bond lengths for the two crystallographically independent PAA donors in $(PAA)_4{\cdot}(F_4TCNQ)$.

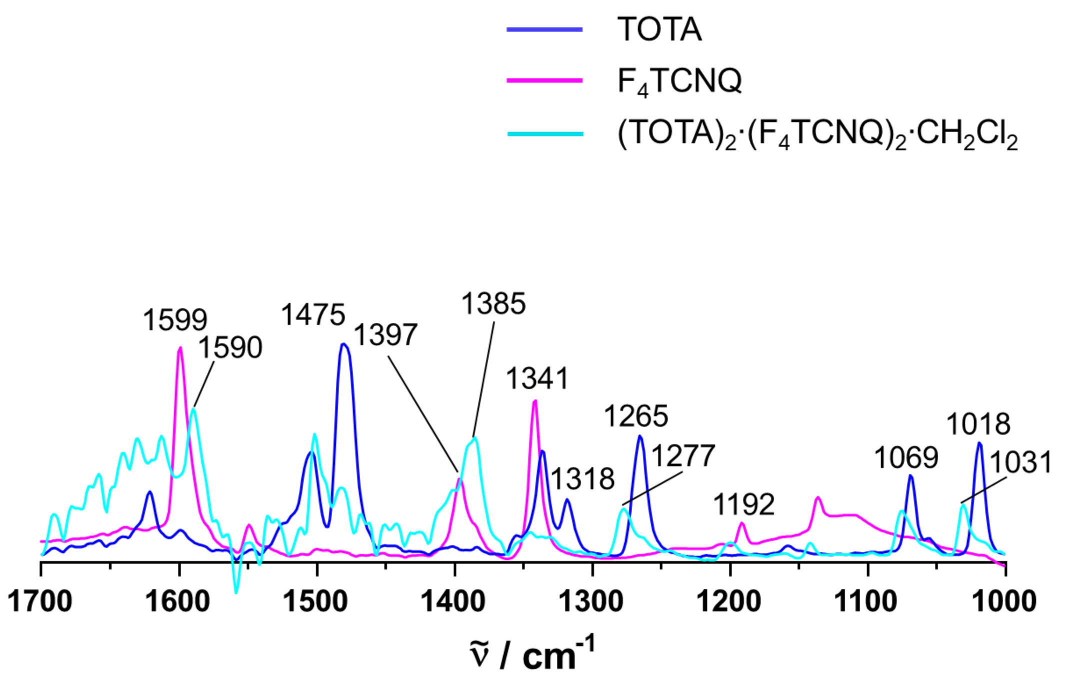

Figure S8. IR spectra for the arene region of the CT compound TOTA·F_4TCNQ in the solid state (as KBr pellet). The blue line represents neutral TOTA, the magenta line the neutral acceptor F_4TCNQ and turquoise line the CT compound.

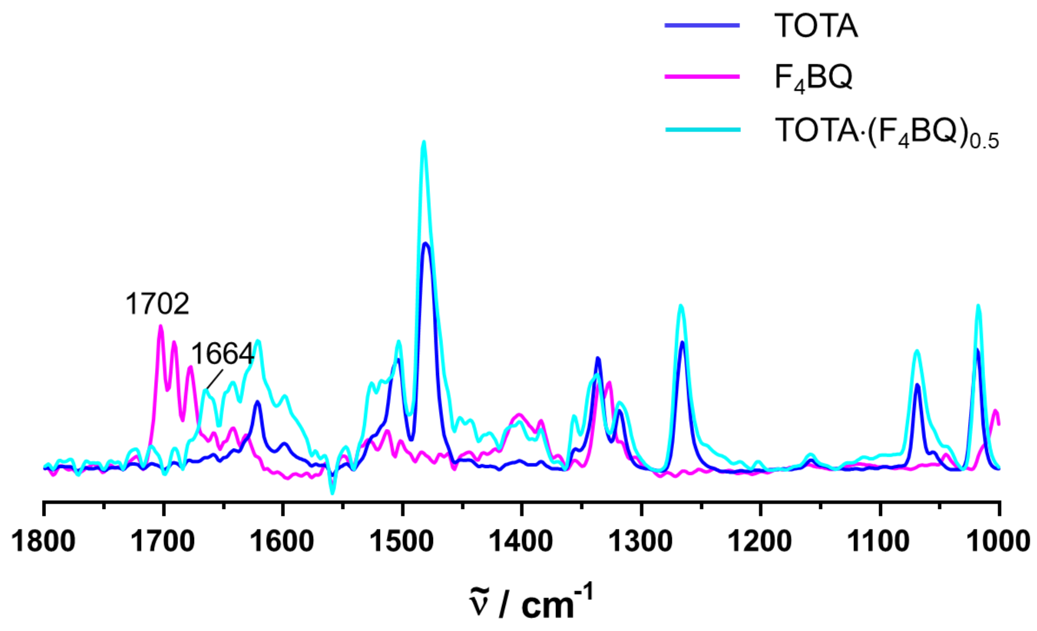

Figure S9. IR spectra for the carbonyl and arene regions of the CT compound $(TOTA)_2 \cdot (F_4BQ)$ in the solid state (as KBr pellet). The blue line represents the neutral TOTA, the magenta line the neutral acceptor F_4BQ and turquoise line the CT compound.

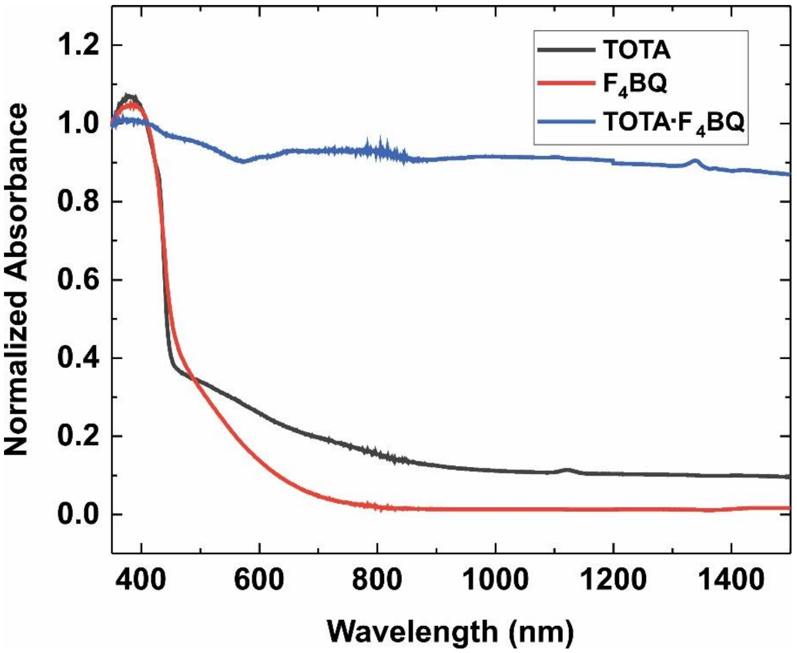

Figure S10. Solid state UV/vis/NIR of TOTA (blue line), F_4BQ (magenta) and $(TOTA)_2·F_4BQ$ (turquoise).

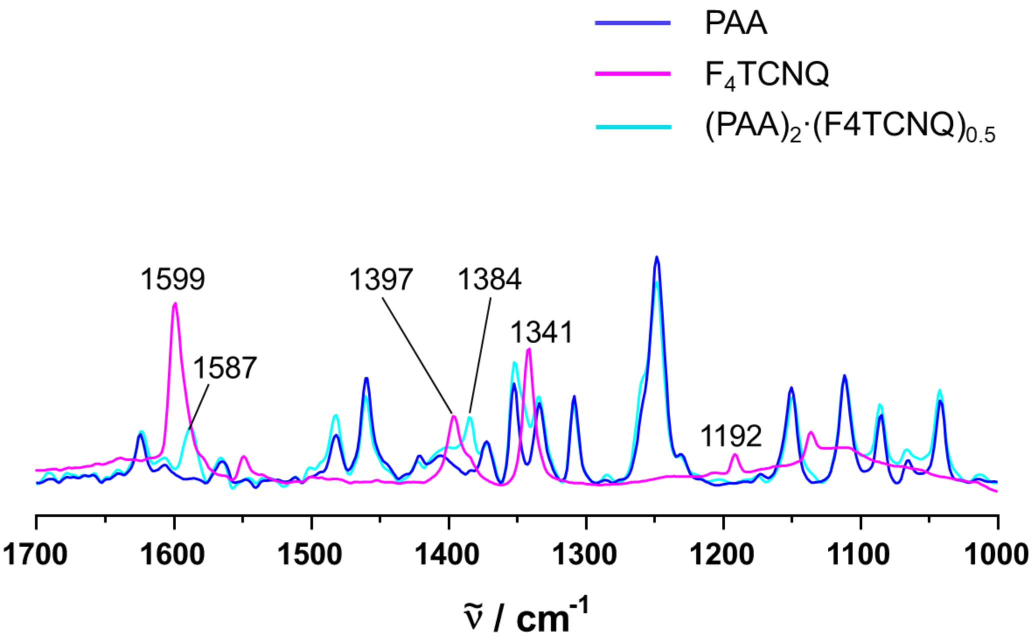

Figure S11. IR spectra for the arene region of the CT compound $(PAA)_4·(F_4TCNQ)$ in the solid state (as KBr pellet). The blue line represents neutral PAA, the magenta line the neutral acceptor F_4TCNQ and the turquoise line the CT compound.

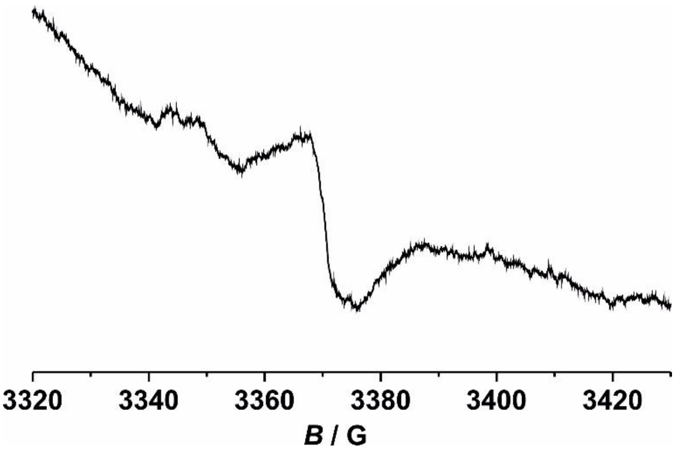

Figure S12. EPR spectrum of a solid sample of $(PAA)_4{\cdot}(F_4TCNQ)$.

Conductivity Studies

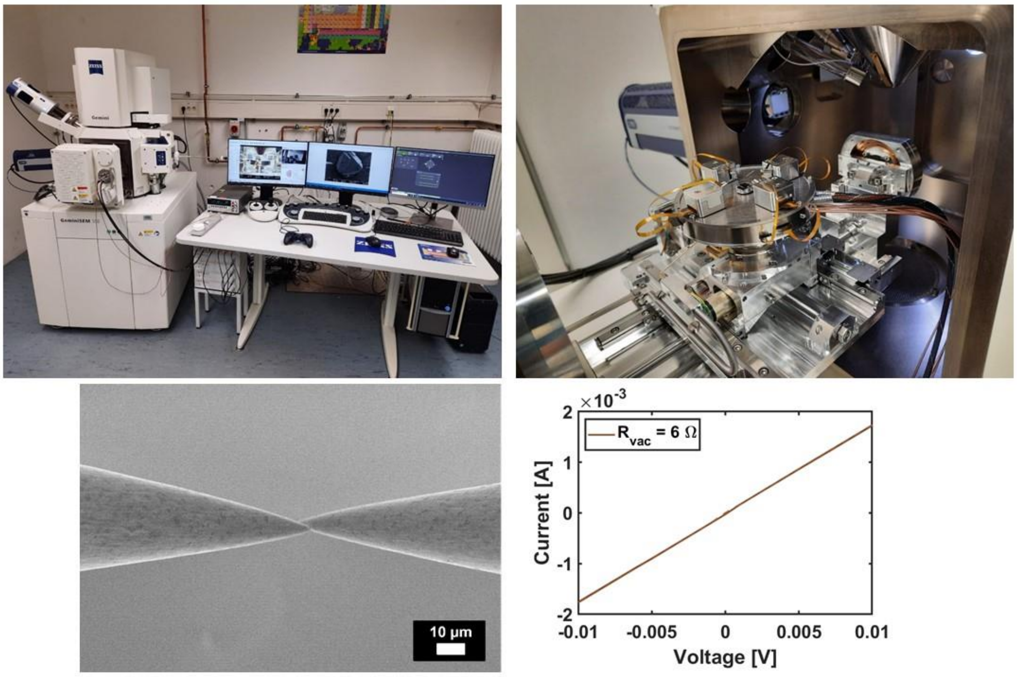

Figure S13. Experimental setup with nanoprobes for measuring the conductivities of solid, crystalline samples of the CT compounds of this study.

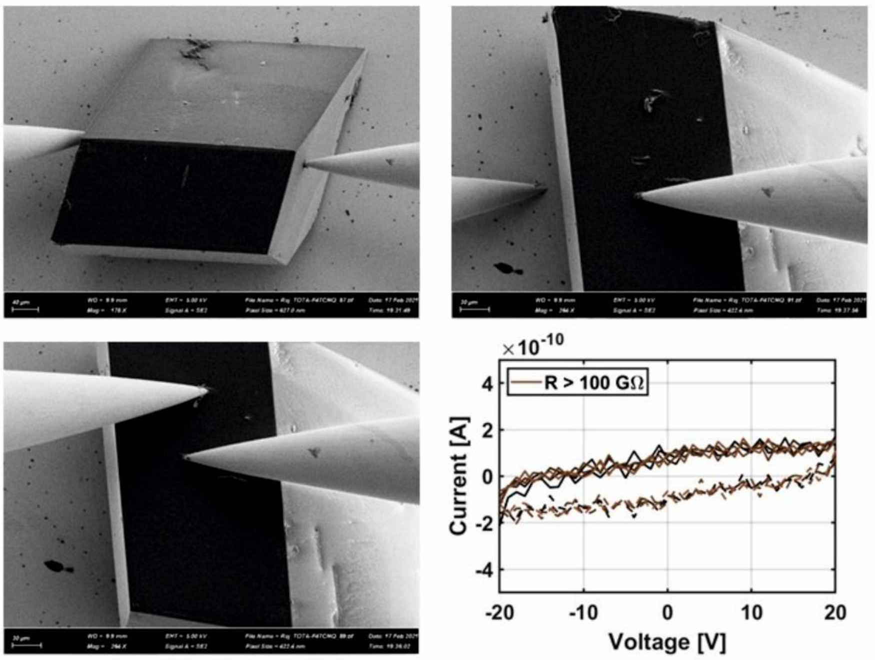

Figure S14. Conductivity measurement of a crystal of $(TOTA)_2{\cdot}(F_4TCNQ)_2{\cdot}CH_2Cl_2$ with nanoprobes under a scanning electron microscope (SEM).

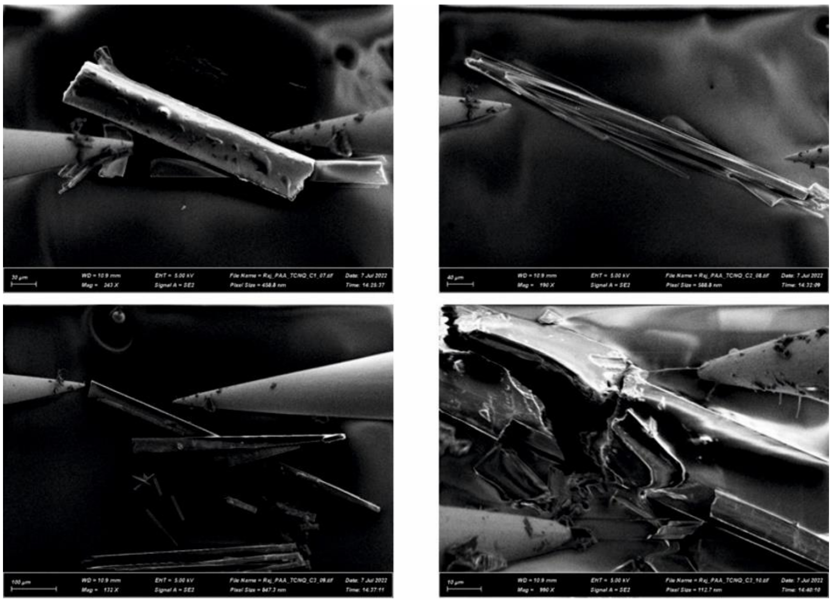

Figure S15. Representative conductivity measurement of a crystal of $(PAA)_4{\cdot}F_4TCNQ$ with nanoprobes under a scanning electron microscope (SEM). No measurable response was recorded.

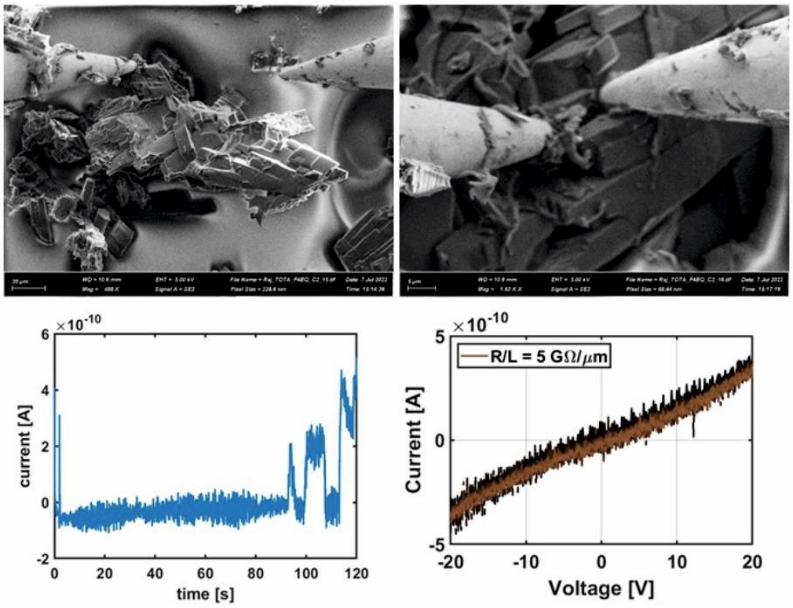

Figure S16. Conductivity measurement of a crystal of $(TOTA)_2{\cdot}F_4BQ$ with nanoprobes under a scanning electron microscope (SEM) at a tip distance of 13 μm.

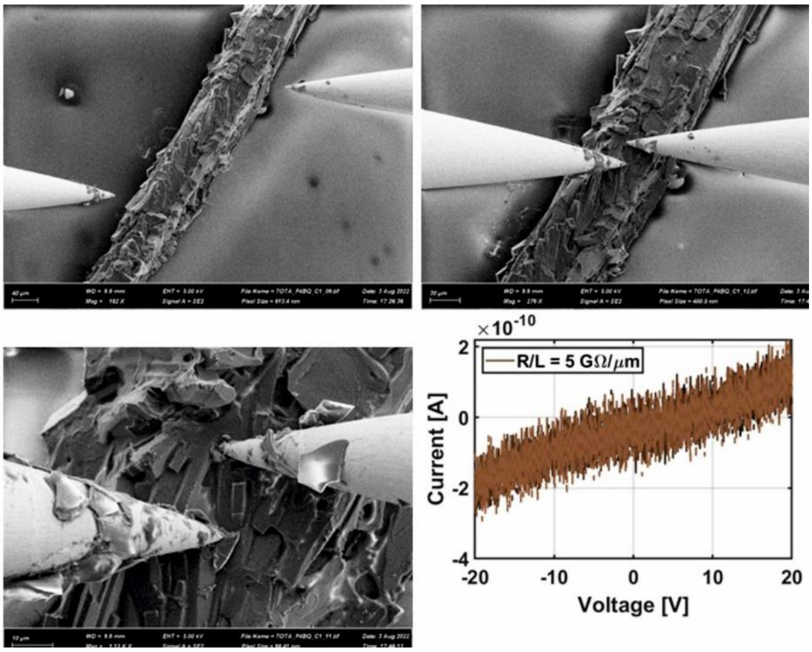

Figure S17. Conductivity measurement of a crystal of $(TOTA)_2{\cdot}F_4BQ$ with nanoprobes under a scanning electron microscope (SEM) at a tip distance of 21 μm.

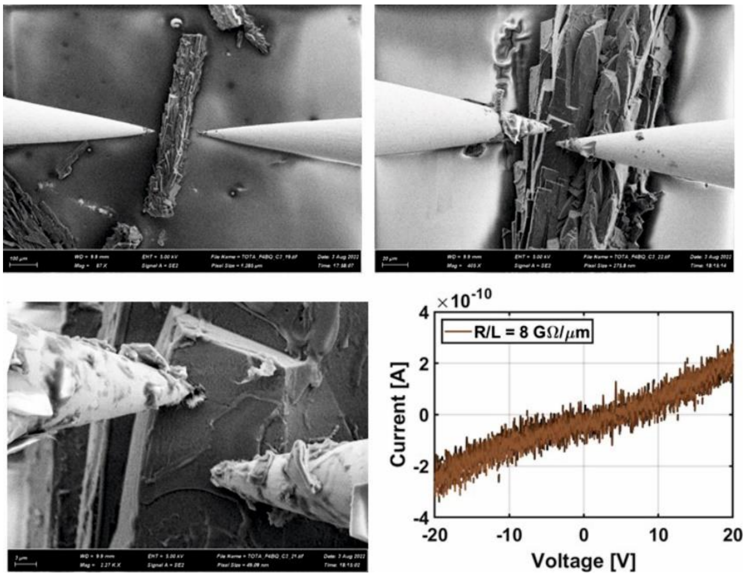

Figure S18. Conductivity measurement of a crystal of $(TOTA)_2{\cdot}F_4BQ$ with nanoprobes under a scanning electron microscope (SEM) at a tip distance of 21 µm.

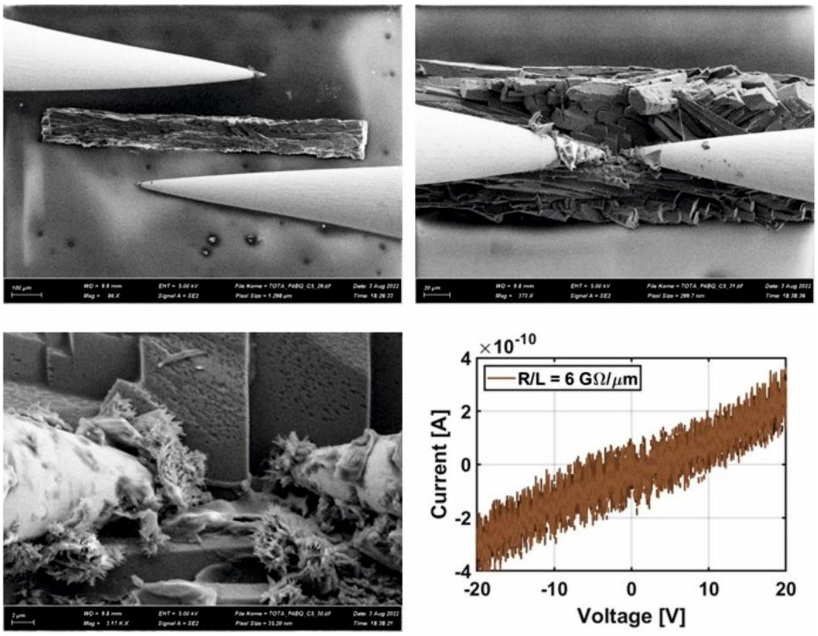

Figure S19. Conductivity measurement of a crystal of $(TOTA)_2{\cdot}F_4BQ$ with nanoprobes under a scanning electron microscope (SEM) at a tip distance of 12 µm.

References

1. M. Kuratsu, M. Kozaki and K. Okada, *Angew. Chem. Int. Ed.*, 2005, **44**, 4056-4058.
2. R. Das, M. Linseis, S. M. Schupp, L. Schmidt-Mende and R. F. Winter, *Chem. Eur. J.*, 2022, **28**, e202104403.
3. O. V. Dolomanov, L. J. Bourhis, R. J. Gildea, J. A. K. Howard and H. Puschmann, *J. Appl. Cryst.*, 2009, **42**, 339-341.
4. G. M. Sheldrick, *Acta Cryst. C*, 2015, **71**, 3-8.
5. C. F. Macrae, I. J. Bruno, J. A. Chisholm, P. R. Edgington, P. McCabe, E. Pidcock, L. Rodriguez-Monge, R. Taylor, J. Van De Streek and P. A. Wood, *J. Appl. Cryst.*, 2008, **41**, 466-470.
